# Downlink Cooperative Broadcast Transmission Based on Superposition Coding in a Relaying System for Future Wireless Sensor Networks

**DOI:** 10.3390/s18061973

**Published:** 2018-06-20

**Authors:** Yang Liu, Guangjie Han, Sulong Shi, Zhengquan Li

**Affiliations:** 1 Jiangsu Provincial Engineerinig Laboratory of Pattern Recognition and Computational Intelligence, Jiangnan University, Wuxi 214122, China; liuyang71354@gmail.com (Y.L.); lzq723@gmail.com (Z.L.); 2 Key Laboratory for Ubiquitous Network and Service Software of Liaoning Province, School of Software, Dalian University of Technology, Dalian 116024, China; 3 Huawei Company, Nanjing 210003, China; shisulong@yeah.net

**Keywords:** broadcast channel, relay channel, repetition coding, superposition coding, successive interference cancellation

## Abstract

This study investigates the superiority of cooperative broadcast transmission over traditional orthogonal schemes when applied in a downlink relaying broadcast channel (RBC). Two proposed cooperative broadcast transmission protocols, one with an amplify-and-forward (AF) relay, and the other with a repetition-based decode-and-forward (DF) relay, are investigated. By utilizing superposition coding (SupC), the source and the relay transmit the private user messages simultaneously instead of sequentially as in traditional orthogonal schemes, which means the channel resources are reused and an increased channel degree of freedom is available to each user, hence the half-duplex penalty of relaying is alleviated. To facilitate a performance evaluation, theoretical outage probability expressions of the two broadcast transmission schemes are developed, based on which, we investigate the minimum total power consumption of each scheme for a given traffic requirement by numerical simulation. The results provide details on the overall system performance and fruitful insights on the essential characteristics of cooperative broadcast transmission in RBCs. It is observed that better overall outage performances and considerable power gains can be obtained by utilizing cooperative broadcast transmissions compared to traditional orthogonal schemes.

## 1. Introduction

In recent years, a wireless sensor network has developed rapidly [1,2,3] and been widely used in many fields, such as meteorology [4,5]. Relaying has been shown to achieve anti-fading capability in the future wireless sensor network [6]. Initial studies on relaying focus on the single source-destination pair scenario and various cooperative transmission protocols have been proposed [7,8]. The most investigated protocols are the amplify-and-forward (AF) and repetition-coded decode-and-forward (DF) protocols with a half-duplex operation, which fit well into existing systems. Despite the diversity gain provided by these relaying strategies, an extra timeslot for message forwarding is required, which leads to a substantial loss [7].

Regarding this, we consider the broadcast channel (BC) where a source node transmits information to a number of users. In BCs, since the source knows the messages of all users, non-orthogonal schemes that transmit multiple user messages simultaneously may reduce the overall consumed bandwidth and exploit the residual degrees of freedom, then potentially provide better performance. Hence, it is necessary to extend relays to BCs (namely RBC) and investigate how the inherent benefits of BCs can be utilized for efficient relaying.

The investigation on the incorporation of RBCs has attracted some interest recently [9,10,11]. Various efficient relaying schemes have been proposed for fading RBCs [12,13,14]. As one of the relaying schemes in the fading RBCs, superposition coding (SupC) can achieve a desirable capacity region by suitable power splitting [15,16,17,18]. By utilizing SupC, the source (as well as the relay) transmits the messages from both users’ messages simultaneously in a single time slot. Two time slots are needed for each transmission round, thus each user is allowed to occupy the full degrees of freedom of the channel and is assured a diversity gain. In the past few years, the performance of SupC exploited in RBCs has been intensively studied.

More generally, they can be categorized into two distinctive types. The first type considers the case where the relay uses the same power splitting factor (PSF) as the source [19,20]. The optimal power allocations have been proposed and the ergodic capacity of this case has been analyzed. The second type focuses on the case where SupC is utilized only by the source with a partial retransmission at the relay [21,22]. The outage probability of this case has been simulated and the efficiency of the proposed scheme has been confirmed.

Basically, the above schemes are constrained with regard to the PSFs and retransmission. Unconstrained schemes in which the source has possibly different PSFs with the relay and the whole retransmission is utilized at the relay may have better performances. However, the interference at the destination node is higher due to the full dependency of the two diversity signals. It is very challenging to analyze the complex signal-to-noise ratio (SNR) of the closed form expression.

In this study, two cooperative broadcast transmission protocols based on SupC in a downlink RBC is proposed By utilizing random dither at the relay, the two diversity signals at the destination are uncorrelated and the SNR is much easier to analyze. Analytical results on the valid region of the PSF pair are provided. The outage events and theoretical outage probabilities of the AF and DF broadcast schemes are calculated and simulated.

The rest of this paper is organized as follows. Section 2 describes the system model of this study and provides the details of the two proposed cooperative broadcast transmission protocols. The outage events of the two proposed schemes are analyzed in Section 3. Section 4 discusses the power gain and corresponding resource allocation problems. Numerical results are provided in Section 5 to demonstrate the comparable performances of the different protocols. Finally, Section 6 concludes the study.

## 2. System Model and Proposed Broadcast Transmission Protocols

### 2.1. System Model

**Figure 1:**
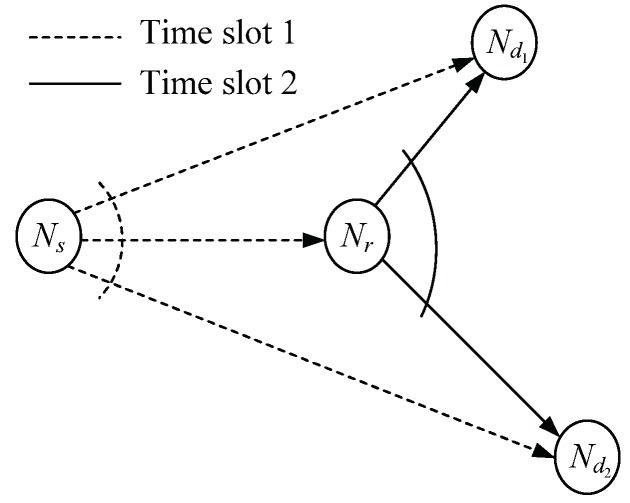
System model of the RBC with two users and one dedicated relay node.

This study investigates the scenario consisting of one source node ($N_{s}$), one relay node ($N_{r}$), and two users ($N_{d_{1}}$,$N_{d_{2}}$), as shown in Figure 1. Each of the two users receives a different message from the source with the help of the relay. We assume that the maximal ratio combining (MRC) detection is used. As in [7,13], the realistic half-duplex constraint is imposed on the nodes, and a time division multiple access (TDMA) system is assumed. Despite the loss in spectral efficiency due to an extra time slot used for relaying, it will be shown that, in multiuser broadcast (downlink) communication scenarios, this drawback can be mitigated by using nonorthogonal transmission strategies. The transmissions between $N_{i}$ and $N_{j}$, i∈{s,r} and $j\in \{r,d_{1},d_{2}\}$, are subject to quasi-static Rayleigh fading and log-distance path loss. We use $h_{ij}$ to denote the complex-valued channel gain of the link between $N_{i}$ and $N_{j}$. The channel gains of the different links are independent. We assume independent and identically distributed (i.i.d), circular symmetric complex-valued additive Gaussian noises at the receiver sides. The details of the AF and DF protocols are given in the following subsections.

### 2.2. Description of Proposed DF Broadcast Transmission Protocol

In the DF protocol considered in this study, two time slots are needed to accomplish a one-round communication at the source with a pair of users, in which the signal delivered by the source, as well as the relay, contains the messages of both users by information superposition. The same is true for the AF protocol. The source transmits the messages of $N_{d_{1}}$ and $N_{d_{2}}$ simultaneously in the first time slot utilizing two-level SupC [19]. After the relay decodes the signal received from the source, the relay re-encodes the recovered messages using the same codebook used at the source subsequently, the codewords are superimposed and forwarded to the users. The signals received at $N_{d_{1}}$, $N_{d_{2}}$, and $N_{r}$ in the first time slot are defined, respectively, as follows:

(1) $$y_{d_{1},1}=h_{sd_{1}}\left(\sqrt{\alpha P_{s}}x_{1}+\sqrt{\bar{\alpha }P_{s}}x_{2}\right)+n_{d_{1},1},$$

(2) $$y_{d_{2},1}=h_{sd_{2}}\left(\sqrt{\alpha P_{s}}x_{1}+\sqrt{\bar{\alpha }P_{s}}x_{2}\right)+n_{d_{2},1},$$

(3) $$y_{r,1}=h_{sr}\left(\sqrt{\alpha P_{s}}x_{1}+\sqrt{\bar{\alpha },P_{s}}x_{2}\right)+n_{r,1}$$

where $x_{s}$ corresponds to the symbol transmitted by the source that contains the unit energy codewords $x_{1}$ and $x_{2}$ ($x_{1}$ and $x_{2}$ contain the messages to be received by $N_{d_{1}}$ and $N_{d_{2}}$, respectively), $P_{s}$ is the transmitted power of the source, and $n_{j,1}$ is the additive white Gaussian noises at nodes $N_{j}$ in the first time slot, where each has variance of $\sigma^{2}=\frac{N_{0}}{2}$ per complex dimension. α is the PSF of the source and indicates the fraction of the power allocated for the transmission of $x_{1}$ with the remainder used for $x_{2}$; $\bar{\alpha }=1-\alpha$. Provided that $x_{1}$ and $x_{2}$ have been successfully decoded by the relay, the users receive in the second time slot

(4) $$y_{d_{1},2}=h_{rd_{1}}\left(\sqrt{\beta P_{r}}x_{1}+\sqrt{\bar{\beta }P_{r}}x_{2}\right)+n_{d_{1},2},$$

(5) $$y_{d_{2},2}=h_{rd_{2}}\left(\sqrt{\beta P_{r}}x_{1}+\sqrt{\bar{\beta }P_{r}}x_{2}\right)+n_{d_{2},2},$$

where $P_{r}$ is the transmitted power of the relay $N_{r}$, $n_{j,2}$ is the additive white Gaussian noise (AWGN) at nodes $N_{j}$ in the second time slot, where each has a variance of $\sigma^{2}=\frac{N_{0}}{2}$ per complex dimension; β is the PSF of the relay; and $\bar{\beta }=1-\beta$.

### 2.3. Description of Proposed AF Broadcast Transmission Protocol

In contrast to the DF protocol, in the AF case, the relay amplifies the received signal (including the noise) by a suitable factor, such that its transmitted power constraint is not affected, and forwards the scaled version to the users in the second time slot. The corresponding received signals by $N_{d_{1}}$, $N_{d_{2}}$, and $N_{r}$ in the first time slot are as described in Equations (1)–(3), respectively. The relay amplifying factor is

(6) $$G=\sqrt{\frac{P_{r}}{P_{s}{\left|h_{sr}\right|}^{2}+N_{0}}}.$$

$N_{d_{1}}$ and $N_{d_{2}}$ receive in the second time slot

(7) $$\begin{array}{cc} y_{d_{1},2} & =h_{rd_{1}}Gy_{r,1}+n_{d_{1},2}\\ & =h_{rd_{1}}Gh_{sr}\left(\sqrt{\alpha P_{s}}x_{1}+\sqrt{\bar{\alpha }P_{s}}x_{2}\right)+\;h_{rd_{1}}Gn_{r,1}+n_{d_{1},2}, \end{array}$$

(8) $$y_{d_{2},2}=h_{rd_{2}}Gh_{sr}\left(\sqrt{\alpha P_{s}}x_{1}+\sqrt{\bar{\alpha }P_{s}}x_{2}\right)+\;h_{rd_{2}}Gn_{r,1}+n_{d_{2},2}.$$

For both the AF and DF broadcast transmission protocols, the receivers combine the signals received from $N_{s}$ and $N_{r}$ using MRC and perform successive interference cancellation (SIC) to recover the messages.

### 2.4. Notations

For notational convenience, we use

(9) $$\gamma_{ij}=P_{i}\frac{|h_{ij}{|}^{2}}{N_{0}}$$

to denote the instantaneous SNR of the link between $N_{i}$ and $N_{j}$, i∈{s,r}, and $j\in \{r,d_{1},d_{2}\}$. In addition, we use

(10) $$\Gamma_{ij}=P_{i}\cdot PL_{ij}$$

to denote the mean value of $\gamma_{ij}$, where $PL_{ij}$ is the path loss of the link from $N_{i}$ to $N_{j}$. It can be easily deduced that the $\gamma_{ij}$s are independent exponentially distributed random variables with parameters $1/\Gamma_{ij}$. The coding rates of $x_{1}$ and $x_{2}$ are denoted by $\tau_{1}$ and $\tau_{2}$ throughout the rest of this paper.

## 3. Outage Analysis of Proposed Cooperative Broadcast Transmission Protocols

### 3.1. Outage Analysis of Proposed DF Broadcast Transmission Protocol

The main feature of a broadcast transmission with respect to an orthogonal transmission is that the messages aimed at isolated users are superimposed before the transmission, hence we have to detect the user messages from a maximum ratio combination of two independent superimposed signals at the receivers, which is much more complicated to analyze. As was stated in the introduction, we assume a pre-fixed decoding order with SIC at the receivers. Without loss of generality, the message of user 2 ($x_{2}$) is decoded first in this study. Although the approach in [23] was proposed and studied in the single-user fading channel scenario, it fits well into multi-user/multi-receiver systems [16,19]. First, as an example, we consider the use of the SIC approach in [23] in the two-user fading BC.

#### 3.1.1. Selection of the PSF in the Two-User Fading Broadcast Channel

We use the same notations and assumptions as for the dedicated RBC, with the exception that there is no relay node. Two channel thresholds $∥h_{1}∥$ and $∥h_{2}∥$ are used to indicate the channel condition required for successful decoding of $x_{1}$ and $x_{2}$, respectively. Since $x_{2}$ (the message of user 2) is decoded first, $∥h_{2}∥$ denotes the bad channel state [16,23], namely

(11) $$\begin{array}{c} |h_{2}\left|\leq \right|h_{1}|. \end{array}$$

In addition, we have the following expressions of the message rates $\tau_{1}$ and $\tau_{2}$, which are related to the channel thresholds and the PDF α:

(12) $$\begin{array}{c} \tau_{2}=\log\left(1+\frac{\bar{\alpha }P_{s}{\left|h_{2}\right|}^{2}}{1+\alpha P_{s}{\left|h_{2}\right|}^{2}}\right), \end{array}$$

(13) $$\begin{array}{c} \tau_{1}=\log\left(1+\alpha P_{s}{\left|h_{1}\right|}^{2}\right). \end{array}$$

It should be noted that the term $\alpha P_{s}{\left|h_{2}\right|}^{2}$ in the denominator of Equation (12) indicates the noise introduced by $x_{1}$ when decoding $x_{2}$. The inequality in Equation (11) implies that a receiver can never decode the message of $N_{d_{1}}$ alone. In other words, the message of $N_{d_{2}}$ is physically degraded to that of $N_{d_{1}}$. After some manipulations, Equations (12) and (13) can be rephrased as

(14) $$\begin{array}{c} P_{s}{\left|h_{2}\right|}^{2}=\frac{2^{\tau_{2}}-1}{1-\alpha 2^{\tau_{2}}},\alpha <\frac{1}{2^{\tau_{2}}}, \end{array}$$

(15) $$\begin{array}{c} P_{s}{\left|h_{1}\right|}^{2}=\frac{2^{\tau_{1}}-1}{\alpha }. \end{array}$$

Combining Equations (11), (14) and (15), a valid range of α is obtained as follows:

(16) $$\alpha \leq \frac{2^{\tau_{1}}-1}{2^{\tau_{1}}2^{\tau_{2}}-1}=\alpha^{max}.$$

The notation $\alpha^{max}$ is used instead of $\frac{2^{\tau_{1}}-1}{2^{\tau_{1}}2^{\tau_{2}}-1}$ throughout the rest of this paper.

The above discussion is for the broadcast system without a relay, in which only a single superimposed signal is received at the destinations. The problem is much more complicated in dedicated RBCs because two superimposed signals are received at the destinations and are combined using MRC (as is assumed in this study). Fortunately, a similar conclusion can be drawn for successive decoding over combined superimposed signals as over a single superimposed signal.

#### 3.1.2. Selection of the PSF in the DF Broadcast Transmission

Now, we proceed to consider the DF protocol for the dedicated RBC. As we described in Section 2, the user messages are transmitted over two consecutive time slots. In the first time slot, the source transmits a superposition of $x_{1}$ and $x_{2}$; the relay and both users listen. The second time slot transmission can be one of three cases depending on whether the relay successfully decodes $x_{1}$ and/or $x_{2}$.

**Case 1**: First, suppose a correct recovery of the messages of both users at the relay, which corresponds to the following event:

(17) $$\left[\frac{\bar{\alpha }P_{s}{\left|h_{sr}\right|}^{2}}{1+\alpha P_{s}{\left|h_{sr}\right|}^{2}}\geq 2^{\tau_{2}}-1\right]⋂\left[\alpha P_{s}{\left|h_{sr}\right|}^{2}\geq 2^{\tau_{1}}-1\right].$$

Here, in Equation (17) (and in Equations (18) and (19), we temporarily use $P_{i}{\left|h_{ij}\right|}^{2}$ instead of its abbreviation $\gamma_{ij}$ for clarity of expression). $N_{d_{1}}$ will receive a superimposed signal transmitted by the source and the relay as described in Equations (1) and (4). In order to decrease the full dependency of the two diversity signals, random dithering is utilized at the relay. Thus, the codewords $x_{1}$ will be replaced by ${\tilde{x}}_{1}$. We can write the outage event for decoding of $x_{2}$ at $N_{d_{1}}$ as:

(18) $$\frac{\bar{\alpha }P_{s}{\left|h_{sd_{1}}\right|}^{2}}{1+\alpha P_{s}{\left|h_{sd_{1}}\right|}^{2}}+\frac{\bar{\beta }P_{r}{\left|h_{rd_{1}}\right|}^{2}}{1+\beta P_{r}{\left|h_{rd_{1}}\right|}^{2}}<2^{\tau_{2}}-1,$$

and the outage of $x_{1}$ provided that $x_{2}$ has already been successfully decoded is

(19) $$\alpha P_{s}|h_{sd_{1}}{|}^{2}+\beta P_{r}{\left|h_{rd_{1}}\right|}^{2}<2^{\tau_{1}}-1.$$

In view of the assumption regarding the decoding order, we hope that the decoding of $x_{2}$ is physically degraded to that of $x_{1}$ in an appropriate sense (The channel realizations that satisfy the successful decoding of $x_{1}$ also meet the condition to decode $x_{2}$.) as in a conventional broadcast transmission, for which the PSF should be suitably designed.

**Theorem** **1.** *Let*

(20) $$\begin{array}{cc} \Phi_{1} & =\left\{\left(\gamma_{1},\gamma_{2}\right):a\gamma_{1}+b\gamma_{2}<2^{\tau_{1}}-1\right\},\\ \Phi_{2} & =\left\{\left(\gamma_{1},\gamma_{2}\right):\frac{\bar{a}\gamma_{1}}{1+a\gamma_{1}}+\frac{\bar{b}\gamma_{2}}{1+b\gamma_{2}}<2^{\tau_{2}}-1\right\}, \end{array}$$

*where a,b∈[0,1], $\bar{a}=1-a$ and $\bar{b}=1-b$, $\gamma_{1}$ and $\gamma_{2}$ are nonnegative random variables. Then, $\Phi_{2}\subseteq \Phi_{1}$ if and only if*

$$a\leq \alpha^{max}\;and\;b\leq \alpha^{max},$$

*where $\alpha^{max}$ is as defined in Equation* (16).

**Proof** **1.** The proof is shown in Appendix A.  ☐

Theorem 1 indicates that, in Case 1, the decoding of $x_{2}$ at each destination is degraded to that of $x_{1}$ only when α and β are smaller than $\alpha^{max}$.

**Case 2**: Then, consider the case that only $x_{2}$ is correctly decoded by the relay. In this case, $x_{2}$ is retransmitted by the relay with full power in the second time slot. The outage events corresponding to $x_{2}$ and $x_{1}$ (conditioned on the successful decoding of $x_{2}$) at $N_{d_{1}}$ are

(21) $$\frac{\bar{\alpha }\gamma_{sd_{1}}}{1+\alpha \gamma_{sd_{1}}}+\gamma_{rd_{1}}<2^{\tau_{2}}-1$$

and

(22) $$\alpha \gamma_{sd_{1}}<2^{\tau_{1}}-1,$$

respectively.

**Case 3**: When the relay fails to recover the messages of both users, only the signal received from the source can be used to decode $x_{1}$ and $x_{2}$ at each destination. The outage event for decoding of $x_{2}$ at $N_{d_{1}}$ is

(23) $$\frac{\bar{\alpha }\gamma_{sd_{1}}}{1+\alpha \gamma_{sd_{1}}}<2^{\tau_{2}}-1.$$

The outage event corresponding to $x_{1}$ (provided that $x_{2}$ was successfully decoded is as described in Equation (22).

**Corollary** **1.** *For the DF broadcast transmission protocol, the decoding of $x_{2}$ at each receiver is degraded to that of $x_{1}$, if and only if*

(24) $$\alpha \leq \alpha^{max}\;and\;\beta \leq \alpha^{max}.$$

**Proof** **2.** Theorem 1 serves as the necessity proof. Hence, we only need to provide the sufficiency proof. For the decoding process at the relay as well as at $N_{d_{1}}$ and $N_{d_{2}}$ in Case 3, the discussion in Section 3.1.1 has proved the degradedness of $x_{2}$ with respect to $x_{1}$ when $\alpha <\alpha^{max}$. The proof of Case 1 is well provided by Theorem 1. For Case 2, we only need to prove that for an arbitrary SNR value of the link between the relay and $N_{d_{1}}$, the SNR (of the link between the source and $N_{d_{1}}$) required for the correct decoding of $x_{2}$ is lower than that required for the decoding of $x_{1}$ (provided that $x_{2}$ has been subtracted from the received signal). The proof is rather straightforward, hence is omitted here for convenience.  ☐

Although the above discussion is focused on the decoding at $N_{d_{1}}$, the same result can be obtained for the decoding at $N_{d_{2}}$.

#### 3.1.3. Outage Probability of the DF Broadcast Transmission Protocol

Unless elsewhere stated, the PSFs are selected according to Equation (24). Since the three outage cases discussed above are disjoint and $\gamma_{sr}$, $\gamma_{sd_{1}}$ and $\gamma_{rd_{1}}$ are mutually independent, we can write the overall outage probability of $N_{d_{1}}$ as

(25) $$\begin{array}{cc} P_{out,1} & \overset{\left(a\right)}{=}\mathrm{Pr}\left\{O_{2,r}^{c}⋂O_{1|2,r}^{c}\right\}\cdot \left(\mathrm{Pr}\left\{O_{2,d_{1};1}\right\}+\mathrm{Pr}\left\{O_{2,d_{1};1}^{c}⋂O_{1|2,d_{1};1}\right\}\right)\\ & +\;\mathrm{Pr}\left\{O_{2,r}^{c}⋂O_{1|2,r}\right\}\cdot \left(\mathrm{Pr}\left\{O_{2,d_{1};2}\right\}+\mathrm{Pr}\left\{O_{2,d_{1};2}^{c}⋂O_{1|2,d_{1};2}\right\}\right)\\ & +\;\mathrm{Pr}\left\{O_{2,r}\right\}\cdot \left(\mathrm{Pr}\left\{O_{2,d_{1};3}\right\}+\mathrm{Pr}\left\{O_{2,d_{1};3}^{c}⋂O_{1|2,d_{1};3}\right\}\right)\\ & \overset{\left(b\right)}{=}\mathrm{Pr}\left\{O_{1|2,r}^{c}\right\}\cdot \mathrm{Pr}\left\{O_{1|2,d_{1};1}\right\}\\ & +\;\mathrm{Pr}\left\{O_{2,r}^{c}⋂O_{1|2,r}\right\}\cdot \mathrm{Pr}\left\{O_{1|2,d_{1};2}\right\}\\ & +\;\mathrm{Pr}\left\{O_{2,r}\right\}\cdot \mathrm{Pr}\left\{O_{1|2,d_{1};3}\right\}\\ & \overset{\left(c\right)}{=}\mathrm{Pr}\left\{O_{1|2,r}^{c}\right\}\cdot \mathrm{Pr}\left\{O_{1|2,d_{1};1}\right\}\\ & +\;\mathrm{Pr}\left\{O_{1|2,r}\right\}\cdot \mathrm{Pr}\left\{O_{1|2,d_{1};2}\right\}, \end{array}$$

where, to save space, we use $O_{2,r}$ to denote the outage event of $x_{2}$ at $N_{r}$, $O_{1|2,r}$ is the outage event of $x_{1}$ at $N_{r}$ provided that $x_{2}$ has already been correctly decoded, $O_{2,d_{1};\theta }$ is the outage event of $x_{2}$ at $N_{d_{1}}$ in Case θ (θ=1,2,3), and $O_{1|2,d_{1};\theta }$ is the outage event of $x_{1}$ at $N_{d_{1}}$ in Case θ provided that $x_{2}$ has already been correctly decoded. In addition, $O^{c}$ denotes the complementary event. $O_{2,r}^{c}$ and $O_{1|2,r}^{c}$ correspond to the left and right sides, respectively, of Equation (17); $O_{2,d_{1};\theta }$ with θ= 1, 2, and 3 are as described in Equations (18), (21) and (23), respectively; $O_{1|2,d_{1};1}$ corresponds to Equation (19), $O_{1|2,d_{1};2}$ and $O_{1|2,d_{1};3}$ are the same and correspond to Equation (22). In Equation (25), (a) is the general expression, (b) follows from the degradedness of $x_{2}$ with respect to $x_{1}$ in the decoding sense, and, in (c), the last two terms of (b) are combined. We further evaluate Equation (25) as in Equation (26) where the calculation of $\psi_{MRC}$ is as stated in Equation (27):

(26) $$\begin{array}{cc} P_{out,1} & =\left[1-\exp\left(-\frac{2^{\tau_{1}}-1}{\alpha \Gamma_{sr}}\right)\right]\cdot \left[1-\exp\left(-\frac{2^{\tau_{1}}-1}{\alpha \Gamma_{sd_{1}}}\right)\right]\\ & +\;\exp\left(-\frac{2^{\tau_{1}}-1}{\alpha \Gamma_{sr}}\right)\cdot \psi_{MRC}\left(\alpha \gamma_{sd_{1}},\beta \gamma_{rd_{1}},\tau_{1}\right), \end{array}$$

where the calculation of $\psi_{MRC}$ is [7,8]

(27) $$\begin{array}{c} \begin{array}{c} \Psi_{MRC}\left(\gamma_{sd},\gamma_{rd},\tau \right)=\\ \left\{\begin{array}{c} 1-\exp\left(\frac{1-2^{\tau }}{\Gamma_{rd}}\right)-\frac{\Gamma_{sd}}{\Gamma_{sd}-\Gamma_{rd}}\exp\left(\frac{1-2^{\tau }}{\Gamma_{sd}}\right)\cdot \left[1-\exp\left(\frac{\Gamma_{sd}-\Gamma_{rd}}{\Gamma_{sd}}\frac{1-2^{\tau }}{\Gamma_{rd}}\right)\right],\;\Gamma_{sd}\neq \Gamma_{rd},\\ 1-\exp\left(\frac{1-2^{\tau }}{\Gamma_{rd}}\right)+\frac{1-2^{\tau }}{\Gamma_{rd}}\exp\left(\frac{1-2^{\tau }}{\Gamma_{sd}}\right),\;\Gamma_{sd}=\Gamma_{rd}. \end{array}\right. \end{array} \end{array}$$

To obtain the outage probability of $N_{d_{2}}$, we consider the outage events first. The outage event of $x_{2}$ at $N_{d_{2}}$ in each of the above-mentioned three cases can be obtained by substituting $\gamma_{sd_{2}}$ and $\gamma_{rd_{1}}$ for $\gamma_{sd_{1}}$ and $\gamma_{rd_{1}}$, respectively, in the corresponding outage events of $x_{2}$ at $N_{d_{1}}$. We do not repeat the similar process as for calculating the outage probability of $N_{d_{1}}$ and directly give the outage probability of $N_{d_{2}}$ as

(28) $$\begin{array}{cc} P_{out,2} & =\mathrm{Pr}\left\{\alpha \gamma_{sr}\geq 2^{\tau_{1}}-1\right\}\cdot \;\mathrm{Pr}\left\{\frac{\bar{\alpha }\gamma_{sd_{2}}}{1+\alpha \gamma_{sd_{2}}}+\frac{\bar{\beta }\gamma_{rd_{2}}}{1+\beta \gamma_{rd_{2}}}<2^{\tau_{2}}-1\right\}\\ & +\;\mathrm{Pr}\left\{\alpha \gamma_{sr}<2^{\tau_{1}}-1\right\}\cdot \mathrm{Pr}\left\{\frac{\bar{\alpha }\gamma_{sr}}{1+\alpha \gamma_{sr}}\geq 2^{\tau_{2}}-1\right\}\cdot \;\mathrm{Pr}\left\{\frac{\bar{\alpha }\gamma_{sd_{2}}}{1+\alpha \gamma_{sd_{2}}}+\gamma_{rd_{2}}<2^{\tau_{2}}-1\right\}\\ & +\;\mathrm{Pr}\left\{\frac{\bar{\alpha }\gamma_{sr}}{1+\alpha \gamma_{sr}}<2^{\tau_{2}}-1\right\}\cdot \;\mathrm{Pr}\left\{\frac{\bar{\alpha }\gamma_{sd_{2}}}{1+\alpha \gamma_{sd_{2}}}<2^{\tau_{2}}-1\right\}\\ & =\exp\left(-\frac{2^{\tau_{1}}-1}{\alpha \Gamma_{sr}}\right)\cdot \;\underset{\psi_{1}\left(\gamma_{sd_{2}},\gamma_{rd_{2}},\alpha ,\beta ,\tau_{2}\right)}{\underset{︸}{\int \int_{B_{1}}f_{sd_{2}}\left(\gamma_{sd_{2}}\right)f_{rd_{2}}\left(\gamma_{rd_{2}}\right)d\gamma_{sd_{2}}d\gamma_{rd_{2}}}}\\ & +\left[\exp\left(-\frac{2^{\tau_{2}}-1}{\left(1-\alpha 2^{\tau_{2}}\right)\Gamma_{sr}}\right)-\exp\left(-\frac{2^{\tau_{1}}-1}{\alpha \Gamma_{sr}}\right)\right]\cdot \;\underset{\psi_{2}\left(\gamma_{sd_{2}},\gamma_{rd_{2}},\alpha ,\tau_{2}\right)}{\underset{︸}{\int \int_{B_{2}}f_{sd_{2}}\left(\gamma_{sd_{2}}\right)f_{rd_{2}}\left(\gamma_{rd_{2}}\right)d\gamma_{sd_{2}}d\gamma_{rd_{2}}}}\\ & +\left[1-\exp\left(-\frac{2^{\tau_{2}}-1}{\left(1-\alpha 2^{\tau_{2}}\right)\Gamma_{sr}}\right)\right]\cdot \left[1-\exp\left(-\frac{2^{\tau_{2}}-1}{\left(1-\alpha 2^{\tau_{2}}\right)\Gamma_{sd_{2}}}\right)\right], \end{array}$$

where

(29) $$\begin{array}{cc} B_{1} & \equiv \left\{\left(\gamma_{sd_{2}},\gamma_{rd_{2}}\right):\frac{\bar{\alpha }\gamma_{sd_{2}}}{1+\alpha \gamma_{sd_{2}}}+\frac{\bar{\beta }\gamma_{rd_{2}}}{1+\beta \gamma_{rd_{2}}}<2^{\tau_{2}}-1\right\},\\ B_{2} & \equiv \left\{\left(\gamma_{sd_{2}},\gamma_{rd_{2}}\right):\frac{\bar{\alpha }\gamma_{sd_{2}}}{1+\alpha \gamma_{sd_{2}}}+\gamma_{rd_{2}}<2^{\tau_{2}}-1\right\}. \end{array}$$

Using the results in Appendix B, we can expand $\psi_{1}$ and $\psi_{2}$ to obtain

(30) $$\begin{array}{cc} \psi_{1}\left(\gamma_{sd_{2}},\gamma_{rd_{2}},\alpha ,\beta ,\tau_{2}\right) & =\mathrm{Pr}\left\{\left(\gamma_{sd_{2}},\gamma_{rd_{2}}\right)\in B_{1}\right\}\;\;\\ & =\int_{0}^{l}f_{rd_{2}}\left(\gamma_{rd_{2}}\right)\int_{0}^{\hat{p}\left(\gamma_{rd_{2}}\right)}f_{sd_{2}}\left(\gamma_{sd_{2}}\right)d\gamma_{sd_{2}}d\gamma_{rd_{2}}\\ & =\int_{0}^{l}\frac{1}{\Gamma_{rd_{2}}}\exp\left(-\frac{\gamma_{rd_{2}}}{\Gamma_{rd_{2}}}\right)\left[1-\exp\left(-\frac{\hat{p}\left(\gamma_{rd_{2}}\right)}{\Gamma_{sd_{2}}}\right)\right]d\gamma_{rd_{2}}, \end{array}$$

(31) $$\begin{array}{cc} \psi_{2}\left(\gamma_{sd_{2}},\gamma_{rd_{2}},\alpha ,\beta ,\tau_{2}\right) & =\mathrm{Pr}\left\{\left(\gamma_{sd_{2}},\gamma_{rd_{2}}\right)\in B_{2}\right\}\;\;\\ & =\int_{0}^{2^{\tau_{2}}-1}f_{rd_{2}}\left(\gamma_{rd_{2}}\right)\int_{0}^{\tilde{p}\left(\gamma_{rd_{2}}\right)}f_{sd_{2}}\left(\gamma_{sd_{2}}\right)d\gamma_{sd_{2}}d\gamma_{rd_{2}}\\ & =\int_{0}^{\tilde{l}}\frac{1}{\Gamma_{rd_{2}}}\exp\left(-\frac{\gamma_{rd_{2}}}{\Gamma_{rd_{2}}}\right)\left[1-\exp\left(-\frac{\tilde{p}\left(\gamma_{rd_{2}}\right)}{\Gamma_{sd_{2}}}\right)\right]d\gamma_{rd_{2}}, \end{array}$$

where in Equations (30) and (31), $\hat{p}\left(\gamma_{rd_{2}}\right)$ and $\tilde{p}\left(\gamma_{rd_{2}}\right)$ are notations as defined in Appendix B and $\tilde{l}$ equals to $2^{\tau_{2}}-1$.

### 3.2. Outage Analysis of Proposed AF Broadcast Transmission Protocol

Since the AF relay always retransmits an amplified version of its observations, no classified discussion is needed for the second time slot transmission. According to Equations (1) and (7) and the amplification gain *G* in Equation (6), the outage event corresponding to the decoding of $x_{2}$ at $N_{d_{1}}$ is

(32) $$\frac{\bar{\alpha }\gamma_{sd_{1}}}{1+\alpha \gamma_{sd_{1}}}+\frac{\bar{\alpha }\gamma_{sr}\gamma_{rd_{1}}}{\alpha \gamma_{sr}\gamma_{rd_{1}}+\gamma_{sr}+\gamma_{rd_{1}}+1}<2^{\tau_{2}}-1$$

and that of $x_{1}$ conditioned on the successful decoding of $x_{2}$ is

(33) $$\alpha \gamma_{sd_{1}}+\frac{\alpha \gamma_{sr}\gamma_{rd_{1}}}{\gamma_{sr}+\gamma_{rd_{1}}+1}<2^{\tau_{1}}-1.$$

For clarity of expression, we introduce the notation

$$\gamma_{AF}=\frac{\gamma_{sr}\gamma_{rd_{1}}}{\gamma_{sr}+\gamma_{rd_{1}}+1}$$

and the outage events in Equations (32) and (33) can be rephrased as

(34) $$\frac{\bar{\alpha }\gamma_{sd_{1}}}{1+\alpha \gamma_{sd_{1}}}+\frac{\bar{\alpha }\gamma_{AF}}{\alpha \gamma_{AF}+1}<2^{\tau_{2}}-1$$

and

(35) $$\alpha \gamma_{sd_{1}}+\alpha \gamma_{AF}<2^{\tau_{1}}-1,$$

respectively.

Note that $\gamma_{AF}$ is the harmonic mean of the two exponential random variables $\gamma_{sr}$ and $\gamma_{rd_{2}}$. It is well recognized in the literature [24,25] that, at high values of $\Gamma_{sr}$ and $\Gamma_{rd_{2}}$, $\gamma_{AF}$ can be approximated by an exponential random variable with the parameter $\frac{1}{\Gamma_{sr}}+\frac{1}{\Gamma_{rd_{2}}}$. Hence, if we are considering the high SNR approximation of the outage behavior, according to Theorem 1 and the outage events defined in Equations (34) and (35), $x_{2}$ is degraded to $x_{1}$ (from the decoding sense) when $\alpha \leq \alpha^{max}$. In fact, this conclusion applies to the whole SNR region because, for all possible values of $\gamma_{sr}$ and $\gamma_{rd_{2}}$, $\gamma_{AF}$ as a whole can be treated as a nonnegative random variable, which coincides with the assumption of Theorem 1. Therefore, with the AF protocol, the decoding of $x_{2}$ is degraded to that of $x_{1}$ if and only if $\alpha \leq \alpha^{max}$. A more systematic proof of this is provided in Appendix C.

In the following, we focus on the case of $0<\alpha \leq \alpha^{max}$. (When α equals to zero, the problem degrades to a communication of the source with $N_{d_{2}}$ only, which is unexpected.) Then, the outage event of $N_{d_{1}}$ is simply the one in Equation (33) and the outage event of $N_{d_{2}}$ is directly obtained as

(36) $$\frac{\bar{\alpha }\gamma_{sd_{2}}}{1+\alpha \gamma_{sd_{2}}}+\frac{\bar{\alpha }\gamma_{sr}\gamma_{rd_{2}}}{\alpha \gamma_{sr}\gamma_{rd_{2}}+\gamma_{sr}+\gamma_{rd_{2}}+1}<2^{\tau_{2}}-1.$$

Following the same line of discussion as in Appendix D and in Section 5.1.2, the outage probabilities of $N_{d_{1}}$ and $N_{d_{2}}$ have the same formulation as that of conventional AF relaying as

(37) $$P_{out,u}=\int_{0}^{l_{u}}\frac{1}{\Gamma_{sd_{1}}}\exp\left(-\frac{\gamma_{sd_{1}}}{\Gamma_{sd_{1}}}\right)\cdot \left(I_{1}+I_{2}+I_{3}\right)d\gamma_{sd_{1}}$$

with $I_{1}$, $I_{2}$, and $I_{3}$ defined in Equation (50)–(52) and u=1,2. For the outage expression of $N_{d_{1}}$, the notations in Equations (37) and (50)–(52) are specified as follows:

(38) $$\begin{array}{cc} u & =1;\\ l_{1} & =\frac{2^{\tau_{1}}-1}{\alpha };\\ m(\gamma_{sd_{1}}) & =\frac{2^{\tau_{1}}-1}{\alpha }-\gamma_{sd_{1}};\\ n(\gamma ) & =\frac{\left(\gamma +1\right)m\left(\gamma_{sd_{1}}\right)}{\gamma -m(\gamma_{sd_{1}})},\gamma =\gamma_{sr},\gamma_{rd_{1}};\\ \gamma^{*} & =m\left(\gamma_{sd_{1}}\right)+\sqrt{{\left[m\left(\gamma_{sd_{1}}\right)\right]}^{2}+m\left(\gamma_{sd_{1}}\right)}, \end{array}$$

and for $N_{d_{2}}$

(39) $$\begin{array}{cc} u & =2;\\ l_{2} & =\frac{2^{\tau_{2}}-1}{1-\alpha 2^{\tau_{2}}};\\ m(\gamma_{sd_{2}}) & =\frac{2^{\tau_{2}}-1-\frac{\bar{\alpha }\gamma_{sd_{2}}}{1+\alpha \gamma_{sd_{2}}}}{\bar{\alpha }-\alpha \left(2^{\tau_{2}}-1-\frac{\bar{\alpha }\gamma_{sd_{2}}}{1+\alpha \gamma_{sd_{2}}}\right)};\\ n(\gamma ) & =\frac{\left(\gamma +1\right)m\left(\gamma_{sd_{2}}\right)}{\gamma -m(\gamma_{sd_{2}})},\gamma =\gamma_{sr},\gamma_{rd_{1}};\\ \gamma^{*} & =m\left(\gamma_{sd_{2}}\right)+\sqrt{{\left[m\left(\gamma_{sd_{2}}\right)\right]}^{2}+m\left(\gamma_{sd_{2}}\right)}. \end{array}$$

## 4. Power Gain and Resource Allocation

In order to evaluate the overall system performance of different schemes, we consider the power gain of the broadcast protocols over the orthogonal schemes. To calculate the power gain, we first need to obtain the minimum overall transmit power required by each scheme such that a given transmission rate pair can be achieved subject to the outage probabilities $P_{out,1}^{th}$ and $P_{out,2}^{th}$ for $N_{d_{1}}$ and $N_{d_{2}},$ respectively. We use $R_{1}$ and $R_{2}$ to denote the effective information rates of $N_{d_{1}}$ and $N_{d_{2}},$ respectively, and $P_{t}$ for the overall transmit power.

**Figure 2:**
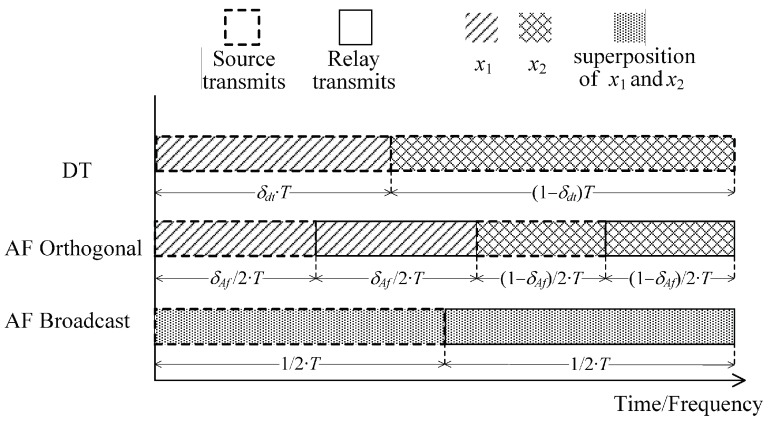
Comparison between the cooperative broadcast transmission and conventional transmission schemes when assigning orthogonal channels to different users.

Note that, for the orthogonal schemes, the channel resources assigned to the transmission of different users’ messages may be different, as illustrated in Figure 2. Only the AF protocols are included for brevity, and the DF counterparts have similar transmission structures. Without loss of generality, the whole channel block per round of transmission is assumed to be 1; $\delta_{dt}$ and $\delta_{af}$ are used to denote the portion of the channel block allocated for the transmission of $x_{1}$ with the remainder for $x_{2}$. In addition, a power allocation between the two users is allowed. For the cooperative broadcast transmission protocols, the optimal power allocation of the power among different users is achieved by simply optimizing the PSFs. We use ζ to denote the ratio of the total power assigned for source transmitting and $\bar{\zeta }=1-\zeta$ for relay forwarding. For the baseline schemes, we reuse the notations α and $\bar{\alpha }$ (β and $\bar{\beta }$) to indicate the percentage of the total source (relay) power used to send $x_{1}$ and $x_{2}$, respectively. The simplified expressions of AF and DF followed by ‘Orthogonal’or ‘Broadcast’ (see Figure 2) will be used instead of their lengthy versions.

Figure 2 shows that the conversion between the effective information rates and the code rates in the DT protocol are

(40) $$\begin{array}{c} \tau_{1}=\frac{R_{1}}{\delta_{dt}},\tau_{2}=\frac{R_{2}}{1-\delta_{dt}}; \end{array}$$

and the practical transmit powers of the source corresponding to $x_{1}$ and $x_{2}$ are

(41) $$\begin{array}{c} P_{s}=\frac{\alpha P_{t}}{\delta_{dt}}\;\mathrm{and}\;P_{s}=\frac{\bar{\alpha }P_{t}}{1-\delta_{dt}}, \end{array}$$

respectively. Similarly, we have for the AF Orthogonal

(42) $$\begin{array}{c} \tau_{1}=\frac{2R_{1}}{\delta_{af}},P_{s}=\frac{2\alpha \zeta P_{t}}{\delta_{af}},P_{r}=\frac{2\beta \bar{\zeta }P_{t}}{\delta_{af}} \end{array}$$

for transmission of $x_{1}$ and

(43) $$\begin{array}{c} \tau_{2}=\frac{2R_{2}}{1-\delta_{af}},P_{s}=\frac{2\bar{\alpha }\zeta P_{t}}{1-\delta_{af}},P_{r}=\frac{2\bar{\beta }\bar{\zeta }P_{t}}{1-\delta_{af}} \end{array}$$

for $x_{2}$. For the AF Broadcast, we have

(44) $$\begin{array}{c} \tau_{1}=2R_{1},\tau_{2}=2R_{2},P_{s}=2\zeta P_{t},P_{r}=2\bar{\zeta }P_{t}. \end{array}$$

Finally, the cases of the DF Orthogonal and DF Broadcast are akin to their AF counterparts and are omitted here for the sake of brevity.

With the above descriptions, the calculation of the minimum overall transmit power is to find the optimal values of α, β, ζ, and $\delta_{dt}$ (or $\delta_{af}$) that minimize $P_{t}$ for given values of $R_{1}$ and $R_{2}$ (or $\tau_{1}$ and $\tau_{2}$). To solve the resource allocation problem analytically is quite difficult due to the complex outage expressions. The situation may be alleviated by using the high SNR approximation of outage behavior. However, the main purpose of this study is to examine the potential advantages of cooperative broadcast transmissions over conventional orthogonal schemes and a specialized investigation in the high SNR regime is beyond the scope of this study. Fortunately, all the variables to be optimized have a valid range of [0,1], which makes it practically feasible to solve the optimization problem by using a numerical search. By using the minimum $P_{t}$ for certain transmission rate pairs of interest ($R_{1}$, $R_{2}$) as well as the target outage probabilities $P_{out,1}^{th}$ and $P_{out,2}^{th}$, the power gain of the broadcast protocols over the baseline schemes can be obtained.

## 5. Numerical Results

**Figure 3:**
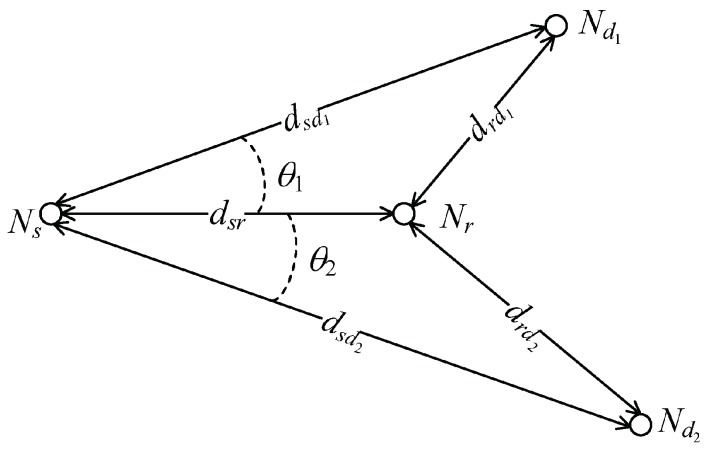
Geometry model of a relay broadcast channel (RBC).

In this section, we present some numerical results to compare the outage performances and power consumptions of the cooperative broadcast transmission protocols with those of the other schemes. We consider a two-dimensional model as in Figure 3, where $\theta_{1}$ is the angle of the line $N_{d_{1}}-N_{s}-N_{r}$, $\theta_{2}$ is the angle of the line $N_{d_{2}}-N_{s}-N_{r}$, and $d_{ij}$ denotes the Euclidean distance between $N_{i}$ and $N_{j}$. Without loss of generality, we use $d_{sd_{2}}$ as a reference distance and consider a number of scenarios with different values of $d_{sd_{1}}$, $d_{sr}$, and $\theta_{1}$, $\theta_{2}$, $d_{rd_{1}}$ and $d_{rd_{2}}$ can be determined by the triangle equalities

(45) $$\begin{array}{cc} {d_{rd_{1}}}^{2} & ={d_{sr}}^{2}+{d_{sd_{1}}}^{2}-2d_{sr}d_{sd_{1}}\cos\left(\theta_{1}\right), \end{array}$$

(46) $$\begin{array}{cc} {d_{rd_{2}}}^{2} & ={d_{sr}}^{2}+{d_{sd_{2}}}^{2}-2d_{sr}d_{sd_{2}}\cos\left(\theta_{2}\right). \end{array}$$

Since a log-distance path loss model is assumed, we have $PL_{ij}=PL_{sd_{2}}\cdot {\left(\frac{d_{ij}}{d_{sd_{2}}}\right)}^{\eta },$ where η is the path loss exponent. Throughout this, we use η=3.75 unless stated otherwise.

### 5.1. Outage Probability of Conventional Relaying with Orthogonal Multiplexing

First, we give the outage probability of conventional relaying with orthogonal multiplexing. The orthogonal AF and DF schemes have the same assumptions as the broadcast transmission protocols in this study, namely half-duplex operation and MRC detection.

#### 5.1.1. Conventional DF Relaying with Orthogonal Multiplexing

The outage probabilities of conventional DF relaying with orthogonal multiplexing at $N_{d_{1}}$ and $N_{d_{2}}$ can be shown as [7,8]

(47) $$\begin{array}{cc} P_{out,1} & =\left[1-\exp\left(-\frac{2^{\tau_{1}}-1}{\Gamma_{sd_{1}}}\right)\right]\cdot \left[1-\exp\left(-\frac{2^{\tau_{1}}-1}{\Gamma_{sr}}\right)\right]\\ & +\;\exp\left(-\frac{2^{\tau_{1}}-1}{\Gamma_{sr}}\right)\cdot \psi_{MRC}\left(\gamma_{sd_{1}},\gamma_{rd_{1}},\tau_{1}\right), \end{array}$$

(48) $$\begin{array}{cc} P_{out,2} & =\left[1-\exp\left(-\frac{2^{\tau_{2}}-1}{\Gamma_{sd_{2}}}\right)\right]\cdot \left[1-\exp\left(-\frac{2^{\tau_{2}}-1}{\Gamma_{sr}}\right)\right]\\ & +\;\exp\left(-\frac{2^{\tau_{2}}-1}{\Gamma_{sr}}\right)\cdot \psi_{MRC}\left(\gamma_{sd_{2}},\gamma_{rd_{2}},\tau_{2}\right). \end{array}$$

#### 5.1.2. Conventional AF Relaying with Orthogonal Multiplexing

The outage probabilities of conventional DF relaying with orthogonal multiplexing at $N_{d_{1}}$ is [7]. (The analytical outage expression is derived in Appendix D)

(49) $$P_{out,1}=\int_{0}^{2^{\tau_{1}}-1}\frac{1}{\Gamma_{sd_{1}}}\exp\left(-\frac{\gamma_{sd_{1}}}{\Gamma_{sd_{1}}}\right)\cdot \left(I_{1}+I_{2}+I_{3}\right)d\gamma_{sd_{1}},$$

where $I_{1}$, $I_{2}$, and $I_{3}$ correspond to the probabilities of the events $A_{1}$, $A_{2}$, and $A_{3}$, respectively, and can be obtained as in (50)–(52):

(50) $$\begin{array}{cc} I_{1} & =1-\int_{m(\gamma_{sd_{1}})}^{\infty }f_{sr}\left(\gamma_{sr}\right)d\gamma_{sr}\cdot \int_{m(\gamma_{sd_{1}})}^{\infty }f_{rd_{1}}\left(\gamma_{rd_{1}}\right)d\gamma_{rd_{1}}\\ & =1-\exp\left(-\frac{m(\gamma_{sd_{1}})}{\Gamma_{sr}}\right)\cdot \exp\left(-\frac{m(\gamma_{sd_{1}})}{\Gamma_{rd_{1}}}\right), \end{array}$$

(51) $$\begin{array}{cc} I_{2} & =\int_{m(\gamma_{sd_{1}})}^{\gamma^{*}\left(\gamma_{sd_{1}}\right)}f_{sr}\left(\gamma_{sr}\right)\cdot \int_{\gamma_{sr}}^{n(\gamma_{sr})}f_{rd_{1}}\left(\gamma_{rd_{1}}\right)d\gamma_{rd_{1}}d\gamma_{sr}\\ & =\int_{m(\gamma_{sd_{1}})}^{\gamma^{*}\left(\gamma_{sd_{1}}\right)}\frac{1}{\Gamma_{sr}}\exp\left(-\frac{\gamma_{sr}}{\Gamma_{sr}}\right)\;\cdot \left[\exp\left(-\frac{\gamma_{sr}}{\Gamma_{rd_{1}}}\right)-\exp\left(-\frac{n(\gamma_{sr})}{\Gamma_{rd_{1}}}\right)\right]d\gamma_{sr}, \end{array}$$

(52) $$\begin{array}{cc} I_{3} & =\int_{m(\gamma_{sd_{1}})}^{\gamma^{*}\left(\gamma_{sd_{1}}\right)}f_{rd_{1}}\left(\gamma_{rd_{1}}\right)\int_{\gamma_{rd_{1}}}^{n(\gamma_{rd_{1}})}f_{sr}\left(\gamma_{sr}\right)d\gamma_{sr}d\gamma_{rd_{1}}\\ & =\int_{m(\gamma_{sd_{1}})}^{\gamma^{*}\left(\gamma_{sd_{1}}\right)}\frac{1}{\Gamma_{rd_{1}}}\exp\left(-\frac{\gamma_{rd_{1}}}{\Gamma_{rd_{1}}}\right)\;\cdot \left[\exp\left(-\frac{\gamma_{rd_{1}}}{\Gamma_{sr}}\right)-\exp\left(-\frac{n(\gamma_{rd_{1}})}{\Gamma_{sr}}\right)\right]d\gamma_{rd_{1}}. \end{array}$$

The outage probability of $N_{d_{2}}$ can be obtained in a straightforward manner by substituting $\tau_{2}$, $\Gamma_{sd_{2}}$, $\Gamma_{rd_{2}}$, $\gamma_{sd_{2}}$, and $\gamma_{rd_{2}}$ for $\tau_{1}$, $\Gamma_{sd_{1}}$, $\Gamma_{rd_{1}}$, $\gamma_{sd_{1}}$, and $\gamma_{rd_{1}}$ in (49)–(52), respectively; the details are omitted here due to space limitations.

### 5.2. Comparison with Other Schemes

**Figure 4:**
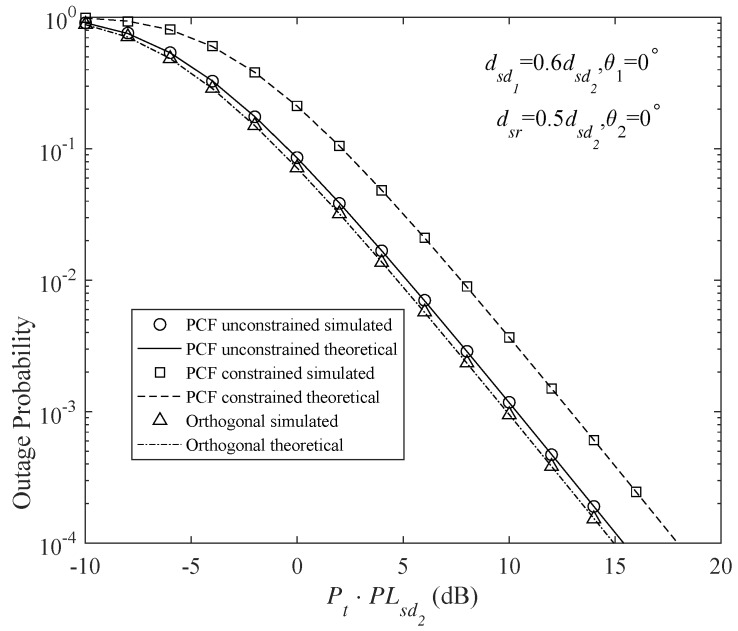
Outage probability of different DF protocols at $N_{d_{1}}$ for code rates $\tau_{1}=\tau_{2}=1$ b/s/Hz ($\tau_{1}=\tau_{2}=2$ b/s/Hz for the orthogonal multiplexing), PCFs of the PCF-constrained protocol $\alpha =0.5\alpha^{max}$ and $\beta =0.5\alpha^{max}$, PCFs of the PCF-unconstrained protocol $\alpha =0.9\alpha^{max}$ and $\beta =0.5\alpha^{max}$, and transmit powers $P_{r}=P_{s}$.

**Figure 5:**
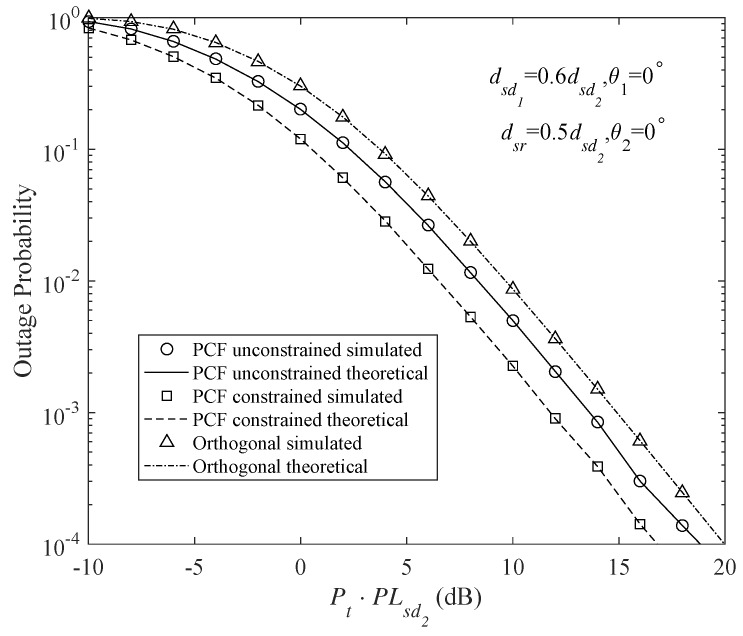
Outage probability of different DF protocols at $N_{d_{2}}$ for code rates $\tau_{1}=\tau_{2}=1$ b/s/Hz ($\tau_{1}=\tau_{2}=2$ b/s/Hz for the orthogonal multiplexing), PCFs of the PCF-constrained protocol $\alpha =0.5\alpha^{max}$ and $\beta =0.5\alpha^{max}$, PCFs of the PCF-unconstrained protocol $\alpha =0.9\alpha^{max}$ and $\beta =0.5\alpha^{max}$, and transmit powers $P_{r}=P_{s}$.

Figure 4 and Figure 5 shows the outage probabilities of the different DF protocols at $N_{d_{1}}$ and $N_{d_{2}}$. These DF protocols include the PSF-unconstrained broadcast, PSF-constrained broadcast (Here, PSF-constrained protocol means that the relay uses the same PSF with the source. However, in the PSF-unconstrained protocol, the PSFs of the source and relay may be different) and orthogonal DF protocols. The source and the relay have the same transmit power, namely $P_{s}=P_{r}$. Since repetition coded relay is assumed for all the schemes, the transmission durations of the source and the relay are equal to each other, hence $P_{t}=\frac{P_{s}+P_{r}}{2}$. It is also assumed that the channel resources (time/frequency) are equally occupied by the users. To have a fair comparison among different protocols, the code rates of the orthogonal cooperative schemes are double those of the broadcast schemes. The analytical results are obtained using the outage expressions in Equations (26), (28), (47) and (48). In addition, Monte Carlo simulations have been provided for these protocols to validate the analytical results. Obviously, the analytical and simulation results match very well.

Figure 4 and Figure 5 clearly indicate that, for the weaker user ($N_{d_{2}}$), a gain of about 3 dB is provided by the PSF-constrained broadcast transmission over the orthogonal scheme, whereas the stronger user ($N_{d_{1}}$) suffers a 3 dB degraded performance. It can be seen that, by appropriately adjusting the PSFs in the PSF-unconstrained broadcast schemes, the outage behavior of $N_{d_{1}}$ can be largely improved. Figure 4 and Figure 5 show that there is only a slight loss in the outage performance of $N_{d_{1}}$ and still a gain of about 1.5 dB is achieved by $N_{d_{2}}$ in the PSF-unconstrained broadcast transmission.

**Figure 6:**
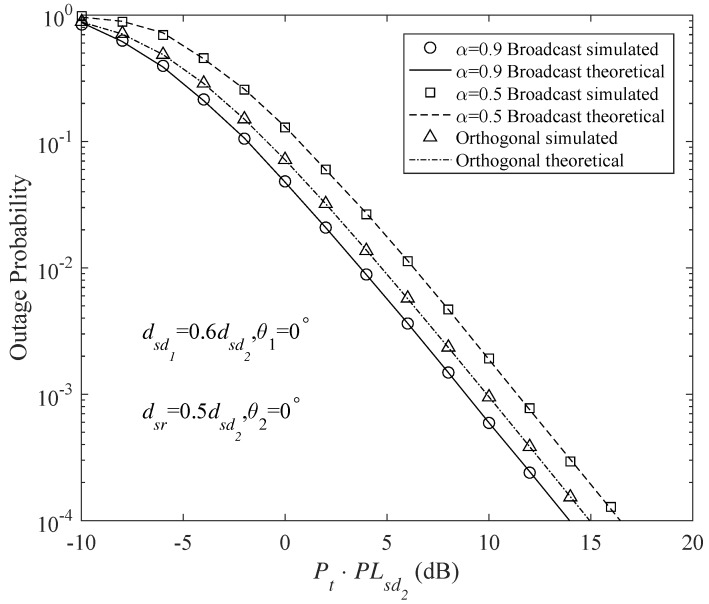
Outage probability of different AF protocols at $N_{d_{1}}$ for code rates $\tau_{1}=\tau_{2}=1$ b/s/Hz ($\tau_{1}=\tau_{2}=2$ b/s/Hz for the orthogonal multiplexing), and transmit powers $P_{r}=P_{s}$.

**Figure 7:**
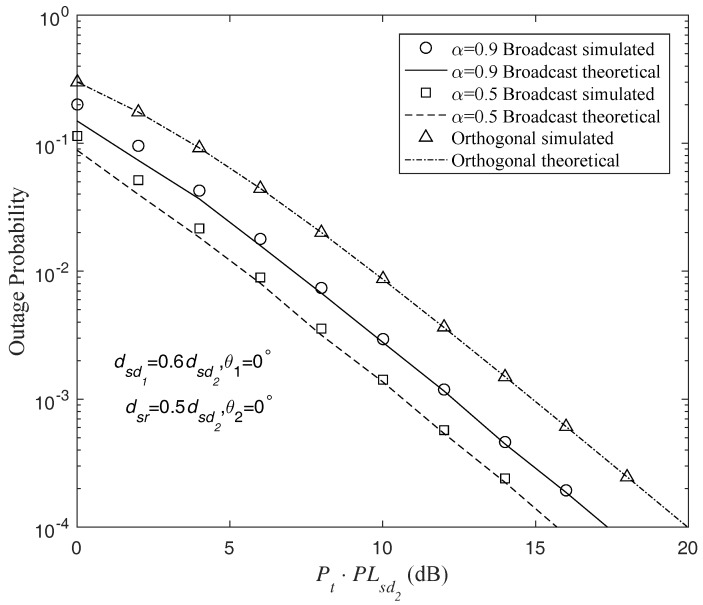
Outage probability of different AF protocols at $N_{d_{2}}$ for code rates $\tau_{1}=\tau_{2}=1$ b/s/Hz ($\tau_{1}=\tau_{2}=2$ b/s/Hz for the orthogonal multiplexing), and transmit powers $P_{r}=P_{s}$.

Similar results can be obtained in the comparisons between the AF broadcast and orthogonal protocols, as shown in Figure 6 and Figure 7. In the AF broadcast scheme with α=0.5, $N_{d_{2}}$ achieves a gain of about 3 dB and $N_{d_{1}}$ suffers a 3dB degraded performance over the orthogonal scheme. However, in the AF broadcast scheme with α=0.9, both ($N_{d_{1}}$) and ($N_{d_{2}}$) obtain enhanced performances compared with the orthogonal scheme. About 2 dB and 1 dB gains are provided by the AF broadcast scheme with α=0.9 for ($N_{d_{1}}$) and ($N_{d_{2}}$), respectively.

Generally, provided that the stronger user’s outage performance satisfies its target error probability, suitable α and β may be selected such that a higher gain can be achieved by the weaker user, which constitutes a better overall system performance.

**Figure 8:**
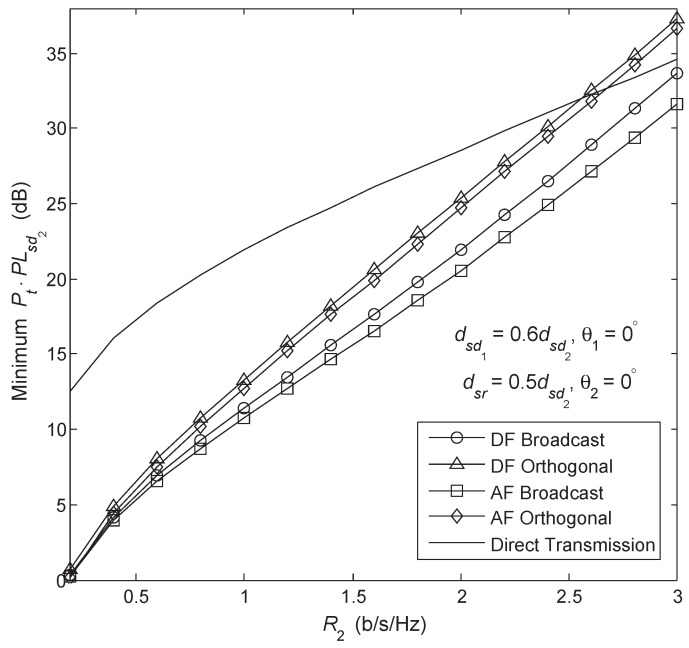
Minimum overall transmit power versus transmission rate with $R_{1}=R_{2}$ and $P_{out}^{th_{1}}=P_{out}^{th_{2}}=0.01$.

Figure 8 shows the case when both users have the same transmission rate and outage constraint with the power and channel resources being optimally allocated such that the overall transmit power $P_{t}$ required to satisfy the target outage probability for each transmission scheme is minimized. The transmission rates $R_{1}$ and $R_{2}$ are as defined in Section 4. The cooperative broadcast schemes generally maintain an advantage over the other collaborative strategies and the power gains become more evident in the higher rate region. As the rate increases, the noncooperative transmission gradually begins to dominates the cooperative methods. This is due to the low spectral efficiency of the repetition coded relay. Although the broadcast schemes are superior from the perspective that the transmission of each user’s message employs the whole time slot, for which $x_{1}$ and $x_{2}$ can have lower code rates and the half-duplex penalty of conventional relaying is alleviated, when we treat $x_{1}$ and $x_{2}$ as a whole, they also suffer from the drawback of the repetition-based relay. Hence, the broadcast transmission schemes are expected to provide the most benefits in relatively (but not extremely) high rate regions.

### 5.3. Effect of Disparity in Channel Qualities and Desired Performances of Users

As it is well known [15], the superiority of broadcast transmissions over those with orthogonal multiplexing is due to the disparity in the user channel qualities. Generally, the degree of disparity is affected by two aspects; one is the distinct channel attenuations suffered by different users’ messages and the other is the disparity in the desired transmission rates and the outage probabilities, which determines the channel quality required by each user to satisfy its target performance.

**Figure 9:**
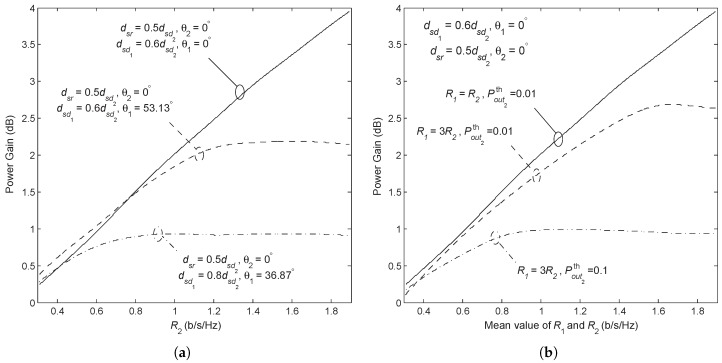
Power gain of AF Broadcast over AF Orthogonal versus rate. In (**a**) $R_{1}=R_{2}$ and $P_{out,1}^{th}=P_{out,2}^{th}=0.01$, the three curves from top to bottom correspond to the disparity in channel qualities of users from large to small, while (**b**) shows the three cases when $N_{d_{2}}$ has the same transmission rate and outage as $N_{d_{1}}$, larger outage probability than $N_{d_{1}}$, and lower transmission rate and larger outage probability than $N_{d_{1}}$.

Figure 9a shows the power gain versus the rate for various network geometries. Only the power gain of the AF Broadcast compared with the AF Orthogonal is shown for simplicity. The case of the DF Broadcast is similar. As it can be expected, as the disparity in the user’s channel qualities decreases, the power gain drops in most of the rate region. The exception occurring in the small rate region can be explained by the fact that the superiority of the AF Broadcast over the AF Orthogonal with regard to spectral efficiency becomes less evident in the lower rate region, whereas a reduction in channel disparity mitigates the disadvantage of the AF Broadcast relative to the AF Orthogonal due to its inability regarding time allocation.

Figure 9b shows the power gain versus the mean transmission rate for various requirements of rate and outage by both users. Requiring a worse quality of service for $N_{d_{2}}$ is equivalent to improving its channel quality relative to $N_{d_{1}}$. Similar trends of the power gain are observed in Figure 9a,b.

**Figure 10:**
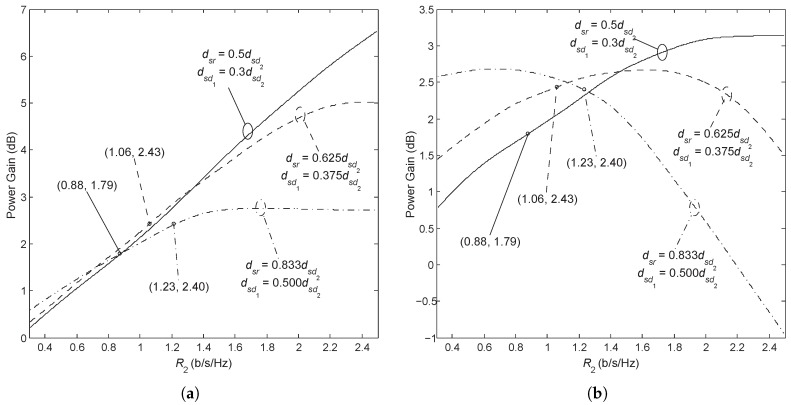
Power gain of AF Broadcast versus the $R_{2}$ with $R_{1}=R_{2}$ and $P_{out,1}^{th}=P_{out,2}^{th}=0.01$. The three curves from top to bottom in (**a**) correspond to the disparity in the channel qualities of users from high to low, all three cases have $\theta_{1}=\theta_{2}=0^{\circ }$. In (**a**), the AF Broadcast is compared with the AF Orthogonal, whereas in (**b**) compared with a mixed AF scheme.

In all of the above-mentioned results, we only include the scenario in which both users’ source-to-destination links are statistically worse than the source-relay link, namely $d_{sr}<d_{sd_{1}}$ and $d_{sr}<d_{sd_{2}}$ (Case 1). A different case occurs when $d_{sr}\geq d_{sd_{1}}$ and $d_{sr}<d_{sd_{2}}$ (Case 2). In Case 2, rather than using AF Orthogonal (DF Orthogonal), a mixed strategy with $N_{d_{1}}$ using a direct transmission and $N_{d_{2}}$ using AF relaying (DF relaying) is preferred in moderately higher rate regions (It should be noted that the mixed schemes are also possible to provide improvement in Case 1, while our purpose is to illustrate the main observations through the selected scenarios, not to cover all situations). Figure 10 shows the power gain of the AF broadcast in comparison with the AF Orthogonal and the mixed AF scheme. It can be seen that the power gain is affected by the transmission rate and the disparity in channel qualities in a similar manner as in Case 1; the only difference is that the power gain (when compared with the mixed scheme) first reaches a peak value as $R_{2}$ increases and then drops until the AF Broadcast is inadequate. The coordinate of the point at which the mixed scheme surpasses the AF Orthogonal has been marked for each channel condition in Figure 10. The conversion points move from right to left as the channel disparity increases, which indicates that, in Case 2 with a large disparity in the channel qualities of users, it is preferred to make the relay serve the weak user only (When the relay forwards the weak user’s message only, the AF (as well as DF) Orthogonal scheme degrades to a mixed scheme.) is better than to use more power to transmit the weak user’s message. The amplified signal received from the relay has a limited contribution to the decoding of $x_{1}$ at $N_{d_{1}}$ considering that the direct link of $N_{d_{1}}$ is better than the source-to-relay link. Moreover, having the relay retransmit $x_{2}$ only largely enhances the successful detection of $x_{2}$ at $N_{d_{2}}$ (as well as at $N_{d_{1}}$); otherwise, much more power has to be used to ensure that $x_{2}$ is received. Again, by comparing Figure 10a,b, we see that the largest power gain is obtained at moderate rate values.

### 5.4. Comparison between AF Broadcast and DF Broadcast

In Figure 4, Figure 5, Figure 6, Figure 7 and Figure 8, we notice that the AF Broadcast provides better system performance than the DF broadcast. Now, we compare the outage event of the AF Broadcast described in Section 3.2 with that of the DF Broadcast described in Section 3.1; three cases can occur. When both users’ messages are fully recovered by the relay, the outage event of the DF Broadcast is a strict subset of the outage event of the AF Broadcast. If the relay fails to decode both $x_{1}$ and $x_{2}$, the outage event of the DF Broadcast covers that of the AF Broadcast. Moreover, when the relay decodes $x_{2}$ only, neither of the outage events of the AF Broadcast and DF Broadcast included in the other. An inherent property of a dedicated-RBC is the high loaded source-to-relay link. Specifically, in this study, the messages of the two users are delivered by the source to the relay through a single source-to-relay link, which increases the chances that the relay fails to recover the user messages. Hence, a no-worse performance is expected from the AF Broadcast than the DF Broadcast, especially when the relay is in close proximity to the destination nodes. However, with a better source-to-relay link, the probability of the successful decoding of $x_{1}$ and $x_{2}$ by the relay is increased and, at the same time, the negative impact of the noise amplification on the performance of the AF Broadcast is reduced. Finally, by averaging over all channel realizations, the possibility of the AF Broadcast to perform worse than the DF Broadcast is quite low.

**Figure 11:**
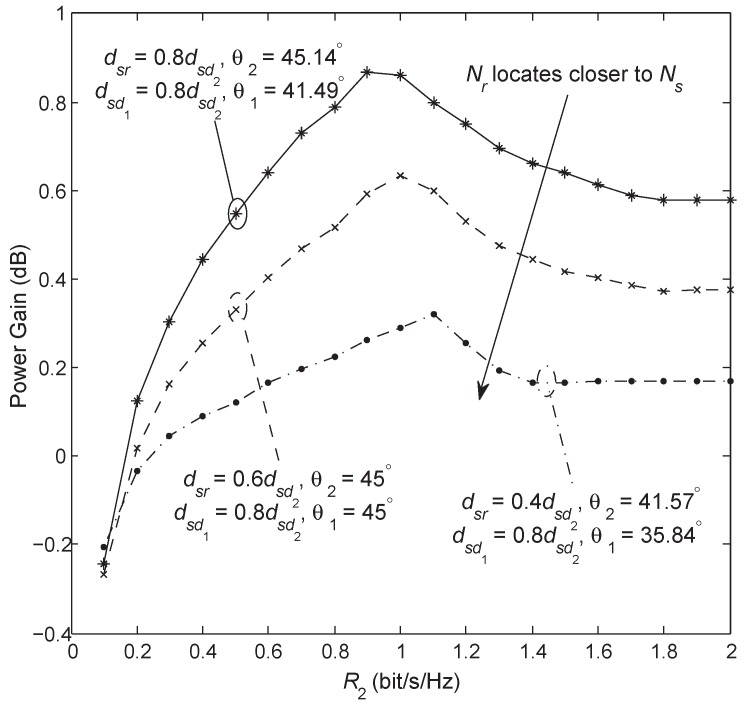
Power gain of AF Broadcast over DF Broadcast versus rate with $R_{1}=R_{2}$ and $P_{out}^{th_{1}}=P_{out}^{th_{2}}=0.01$.

Figure 11 shows the power gain of the AF Broadcast over the DF Broadcast. For comparison purposes, we choose $\theta_{1}$ and $\theta_{2}$ such that the three cases have the same values of $d_{sd_{1}}$, $d_{sd_{2}}$, $d_{rd_{1}}$, and $d_{rd_{2}}$. It can be seen that the AF Broadcast has a better performance for most of the cases and performs slightly worse than the DF Broadcast only in the low-rate regime. The power gain decreases with increasing proximity of the relay to the source.

## 6. Conclusions

In this study, two cooperative broadcast transmission protocols have been considered for the two-user dedicated RBC. By utilizing SupC, the messages of multiple users can be conveyed simultaneously over the same channel block, and by using a portion of the power used for the transmission of each user’s message, a trade-off between the users’ performances is achieved. We have shown that the bad user’s outage behavior can be considerably improved with by a slight increase in the outage probability of the good user, which constitutes a better overall system performance.

The numerical results of a number of scenarios of interest demonstrated that the investigated broadcast transmission strategies generally provide better performances. In addition, the power gain achieved by the cooperative broadcast transmission is largely affected by the level of disparity in the channel qualities and in the quality-of-service requirements of the users, which implies that it is nontrivial to determine the target of cooperation in practical applications; this is an interesting subject for future research. Moreover, it was observed that the broadcast schemes are advantageous in the low to moderate rate regions and provide the furthest gain at certain moderate rates. The comparison between the AF and DF broadcast transmission protocols indicate that a good source-to-relay link is more crucial for the dedicated-RBC than for the conventional relay systems when a regenerative relay is used.

There is additional complexity associated with our cooperative broadcast schemes. First, the utilization of SupC makes the decoding at the relay and at the good user slightly more complex, compared to applications without SupC. In addition, a user pairing procedure is needed prior to the initiation of the communication when applied in a system with more users. Despite the significant improvement provided by our schemes, more efficient protocols should be explored in the future. Furthermore, there are many channel circumstances in addition to those considered in this study that warrant further investigation. Inspired by the results described in Section 5.3 and Section 5.4, more comprehensive studies on the impact of the geometry and quality-of-service requirements on the comparable performances of different protocols are needed to provide guidance for practical applications. 

## Appendix A. Proof of Theorem 1

To proceed, we introduce two auxiliary sets $\Theta_{1}$ and $\Theta_{1}$, which are functions of ω and are defined as follows:

$$\Theta_{1}\left(\omega \right)=\left\{\gamma :\omega \gamma <t_{1}\right\},\Theta_{2}\left(\omega \right)=\left\{\gamma :\frac{\bar{\omega }\gamma }{1+\omega \gamma }<t_{2}\right\},$$

where ω∈[0,1], γ indicates any nonnegative random variables, and $t_{1},t_{2}\geq 0$. It can be simply verified that $\Theta_{2}\subseteq \Theta_{1}$ if and only if $w\leq \frac{t_{1}}{\left(t_{1}+1\right)\left(t_{2}+1\right)-1}$. In the following, we use $\hat{t}$ to denote $\frac{t_{1}}{\left(t_{1}+1\right)\left(t_{2}+1\right)-1}$. Then, we consider the following two sets:

(A1) $$\begin{array}{cc} \Phi_{1} & =\left\{\left(\gamma_{1},\gamma_{2}\right):a\gamma_{1}+b\gamma_{2}<t_{1}\right\},\\ \Phi_{2} & =\left\{\left(\gamma_{1},\gamma_{2}\right):\frac{\bar{a}\gamma_{1}}{1+a\gamma_{1}}+\frac{\bar{b}\gamma_{2}}{1+b\gamma_{2}}<t_{2}\right\}. \end{array}$$

When $a>\hat{t}$, it is obvious that $\Theta_{2}\left(a\right)⊈\Theta_{1}\left(a\right)$. Then, by taking $\gamma_{2}=0$ for $\Phi_{1}$ and $\Phi_{2}$, we obtain $\Phi_{2}⊈\Phi_{1}$. The same is true when $b>\hat{t}$. Hence, it is necessary for *a* and *b* to be no larger than $\hat{t}$ such that $\Phi_{2}\subseteq \Phi_{1}$.

The regions of ($\gamma_{1}$, $\gamma_{2}$) defined by $\Phi_{1}$ and $\Phi_{2}$ can be equivalently expressed as in Equations (A3) and (A4); on top of the next page, where μ and ν are auxiliary variables. Suppose $0<\alpha \leq \hat{t}$. Then, for arbitrarily fixed μ, μ∈[0,1], $\Phi_{1}$ in Equation (A3) has the following equivalent expression:

$$\Phi_{1}=\underset{\Phi_{1a}}{\underset{︸}{\left\{\left(\gamma_{1},\gamma_{2}\right):a\gamma_{1}<\mu t_{1}\right\}}}⋂\underset{\Phi_{1b}}{\underset{︸}{\left\{\left(\gamma_{1},\gamma_{2}\right):b\gamma_{2}<\nu t_{1}\right\}}},$$

where ν=1−μ. For the same μ and ν, $\Phi_{2}$ in Equation (A4) has the following equivalent expression:

(A2) $$\begin{array}{c} \Phi_{2}=\underset{\Phi_{2a}}{\underset{︸}{\left\{\left(\gamma_{1},\gamma_{2}\right):\frac{\bar{a}\gamma_{1}}{1+a\gamma_{1}}<\mu t_{2}\right\}}}⋂\underset{\Phi_{2b}}{\underset{︸}{\left\{\left(\gamma_{1},\gamma_{2}\right):\frac{\bar{b}\gamma_{2}}{1+b\gamma_{2}}<\nu t_{2}\right\}}}. \end{array}$$

It can be simply verified that when $a,b\leq \hat{t}$, $\Phi_{2a}\subseteq \Phi_{1a}$ and $\Phi_{2b}\subseteq \Phi_{1b}$, based on which we definitely have {$\Phi_{2a}⋂\Phi_{2b}\}\subseteq \left\{\Phi_{1a}⋂\Phi_{2b}\right\}$. Thus, $a,b\leq \hat{t}$ is a sufficient condition for $\Phi_{2}$ to be a subset of $\Phi_{1}$.

Recall that $2^{\tau_{1}}-1\geq 0$ and $2^{\tau_{2}}-1\geq 0$, Theorem 1 is proved:

(A3) $$\begin{array}{cc} \Phi_{1} & =\left\{\left(\gamma_{1},\gamma_{2}\right):a\gamma_{1}<\mu t_{1},b\gamma_{2}<\nu t_{1},0\leq \mu \leq 1,\nu =1-\mu \right\}, \end{array}$$

(A4) $$\begin{array}{cc} \Phi_{2} & =\left\{\left(\gamma_{1},\gamma_{2}\right):\frac{\bar{a}\gamma_{1}}{1+a\gamma_{1}}<\mu t_{2},\frac{\bar{b}\gamma_{2}}{1+b\gamma_{2}}<\nu t_{2},0\leq \mu \leq 1,\nu =1-\mu \right\}. \end{array}$$

## Appendix B. Characterization of $B_{1}$ and $B_{2}$

We characterize the sets $B_{1}$ and $B_{2}$, such that the integrals $\psi_{1}$ and $\psi_{2}$ can be directly calculated. First, we consider $B_{1}$ as defined in Equation (29), which can be equivalently expressed as in Equation (A5):

(A5) $$\begin{array}{c} B_{1}\equiv \left\{\begin{array}{c} \left\{\left(\gamma_{sd_{2}},\gamma_{rd_{2}}\right):\frac{\bar{\alpha }\gamma_{sd_{2}}}{1+\alpha \gamma_{sd_{2}}}<2^{\tau_{2}}-1-\frac{\bar{\beta }\gamma_{rd_{2}}}{1+\beta \gamma_{rd_{2}}},\gamma_{rd_{2}}<\frac{2^{\tau_{2}}-1}{1-\beta 2^{\tau_{2}}}\right\},\beta <\frac{1}{2^{\tau_{2}}},\\ \left\{\left(\gamma_{sd_{2}},\gamma_{rd_{2}}\right):\frac{\bar{\alpha }\gamma_{sd_{2}}}{1+\alpha \gamma_{sd_{2}}}<2^{\tau_{2}}-1-\frac{\bar{\beta }\gamma_{rd_{2}}}{1+\beta \gamma_{rd_{2}}}\right\},\;\;\beta \geq \frac{1}{2^{\tau_{2}}}. \end{array}\right. \end{array}$$

For brevity, we introduce the following notation:

(A6) $$p\left(\gamma_{rd_{2}}\right)=2^{\tau_{2}}-1-\frac{\bar{\beta }\gamma_{rd_{2}}}{1+\beta \gamma_{rd_{2}}}.$$

It can be simply verified that $p(\gamma_{rd_{2}})>0$ with the constraint on $\gamma_{rd_{2}}$ as stated in Equation (A5). The first constraint in Equation (A5) can be equivalently expressed as

(A7) $$\left[\bar{\alpha }-\alpha p\left(\gamma_{rd_{2}}\right)\right]\gamma_{sd_{2}}<p\left(\gamma_{rd_{2}}\right),$$

which falls into two cases based on whether $\bar{\alpha }$ is larger than $\alpha p(\gamma_{rd_{2}})$. When $\bar{\alpha }\leq \alpha p\left(\gamma_{rd_{2}}\right)$, Equation (A7) establishes for all valid values of $\gamma_{sd_{2}}$.

When $\bar{\alpha }>\alpha p\left(\gamma_{rd_{2}}\right)$, Equation (A7) degrades to

(A8) $$\gamma_{sd_{2}}<\frac{p(\gamma_{rd_{2}})}{\bar{\alpha }-\alpha p\left(\gamma_{rd_{2}}\right)}.$$

Now, we consider the condition $\bar{\alpha }\leq \alpha p\left(\gamma_{rd_{2}}\right)$, which can be rephrased as

(A9) $$\left[\bar{\beta }-\beta \left(2^{\tau_{2}}-\frac{1}{\alpha }\right)\right]\gamma_{rd_{2}}\leq 2^{\tau_{2}}-\frac{1}{\alpha }.$$

It is obvious from Equation (A9) when $\alpha <\frac{1}{2^{\tau_{2}}}$, $\bar{\alpha }\leq \alpha p\left(\gamma_{rd_{2}}\right)$ is always false; when $\alpha \geq \frac{1}{2^{\tau_{2}}}$, if $\bar{\beta }-\beta \left(2^{\tau_{2}}-\frac{1}{\alpha }\right)\leq 0$ (i.e. $\beta \geq \frac{1}{2^{\tau_{2}}+1-1/\alpha })$, $\bar{\alpha }\leq \alpha p\left(\gamma_{rd_{2}}\right)$ establishes for all valid $\gamma_{rd_{2}}$, else $\bar{\alpha }\leq \alpha p\left(\gamma_{rd_{2}}\right)$ only when

(A10) $$\gamma_{rd_{2}}\leq \frac{2^{\tau_{2}}-\frac{1}{\alpha }}{\bar{\beta }-\beta \left(2^{\tau_{2}}-\frac{1}{\alpha }\right)}.$$

Based on all above discussions and the fact that $\alpha \leq \alpha^{max}$, $\beta \leq \alpha^{max}$, and $\alpha^{max}\leq \frac{1}{2^{\tau_{2}}}$ (the equality is valid only when $\tau_{2}$ equals to zero), we have, for the case of interest of $\tau_{2}>0$ (When $\tau_{2}=0$, there is no message to be transmitted to $N_{d_{2}}$, and the PSFs should be assigned such that α=1 and β=1, namely all the power are allocated for transmission of $N_{d_{1}}$’s message; as a result, the BRC degrades to a conventional relay channel. Similarly, in Appendix C, we have the assumptions that α>0, $\tau_{2}>0$, and $\tau_{1}>0$.), that

(A11) $$B_{1}\equiv \left\{\left(\gamma_{sd_{2}},\gamma_{rd_{2}}\right):\gamma_{sd_{2}}<\hat{p}\left(\gamma_{rd_{2}}\right),\gamma_{rd_{2}}<l\right\},$$

where, for convenience, we have used the following notations:

(A12) $$\hat{p}\left(\gamma_{rd_{2}}\right)=\frac{p(\gamma_{rd_{2}})}{\bar{\alpha }-\alpha p\left(\gamma_{rd_{2}}\right)},\;l=\frac{2^{\tau_{2}}-1}{1-\beta 2^{\tau_{2}}}.$$

Then, we consider $B_{2}$ as defined in Equation (29). In fact, $B_{2}$ is a special case of $B_{1}$ with β=0, hence $B_{2}$ can be equivalently expressed as

(A13) $$B_{2}\equiv \left\{\left(\gamma_{sd_{2}},\gamma_{rd_{2}}\right):\gamma_{sd_{2}}<\tilde{p}\left(\gamma_{rd_{2}}\right),\gamma_{rd_{2}}<2^{\tau_{2}}-1\right\},$$

where

(A14) $$\tilde{p}\left(\gamma_{rd_{2}}\right)=\frac{2^{\tau_{2}}-1-\gamma_{rd_{2}}}{\bar{\alpha }-\alpha \left(2^{\tau_{2}}-1-\gamma_{rd_{2}}\right)}.$$

## Appendix C. Degradeness Condition in the AF Protocol

We consider the outage events in Equations (32) and (33), and denote them using the notations $O_{2,d_{1}}$ and $O_{1|2,d_{1}},$ respectively. We focus on the scenario when α>0, $\tau_{2}>0$, and $\tau_{1}>0$, and the problem falls into two cases based on whether α is smaller than $\frac{1}{2^{\tau_{2}}}$. First, we consider the case of $\alpha \geq \frac{1}{2^{\tau_{2}}}$. It can be simply verified that

$$\frac{\bar{\alpha }\gamma_{sd_{1}}}{1+\alpha \gamma_{sd_{1}}}<2^{\tau_{2}}-1$$

is a certain event by considering its equivalent event

$$\left(1-\alpha 2^{\tau_{2}}\right)\gamma_{sd_{1}}<2^{\tau_{2}}-1.$$

Thus, the outage event $O_{2,d_{1}}$ can be equivalently expressed as

(A15) $$\begin{array}{c} \left[\mathrm{all}\mathrm{valid}\;\gamma_{sd_{1}}\right]⋂\left[\frac{\bar{\alpha }\gamma_{sr}\gamma_{rd_{1}}}{\alpha \gamma_{sr}\gamma_{rd_{1}}+\gamma_{sr}+\gamma_{rd_{1}}+1}<2^{\tau_{2}}-1-\frac{\bar{\alpha }\gamma_{sd_{1}}}{1+\alpha \gamma_{sd_{1}}}\right]. \end{array}$$

In addition, the outage event $O_{1|2,d_{1}}$ has the following equivalent expression

(A16) $$\left[\gamma_{sd_{1}}<\frac{2^{\tau_{1}}-1}{\alpha }\right]⋂\left[\frac{\gamma_{sr}\gamma_{rd_{1}}}{\gamma_{sr}+\gamma_{rd_{1}}+1}<\frac{2^{\tau_{1}}-1}{\alpha }-\gamma_{sd_{1}}\right].$$

Definitely, from Equations (A15) and (A16), $O_{2,d_{1}}$ is not a subevent of $O_{1|2,d_{1}}$. Hence, $x_{2}$ is not degraded to $x_{1}$ when $\alpha \geq \frac{1}{2^{\tau_{2}}}$. Now, we consider the case of $0<\alpha <\frac{1}{2^{\tau_{2}}}$. Following the same line of discussion as in Appendix D and Section 5.1.2, the outage events $O_{1|2,d_{1}}$ can be decomposed and rephrased as

(A17) $$\begin{array}{c} \left\{\gamma_{sd_{1}}<l_{1}\right\}⋂\{\left[\gamma_{sr}\leq m\left(\gamma_{sd_{1}}\right)⋃\gamma_{rd_{1}}\leq m\left(\gamma_{sd_{1}}\right)\right]\;\\ \;⋃\left[\left(m\left(\gamma_{sd_{1}}\right)<\gamma_{sr}<\gamma^{*}\right)⋂\left(\gamma_{sr}<\gamma_{rd_{1}}<n\left(\gamma_{sr}\right)\right)\right]\\ \;⋃\left[\left(m\left(\gamma_{sd_{1}}\right)<\gamma_{rd_{1}}<\gamma^{*}\right)⋂\left(\gamma_{rd_{1}}\leq \gamma_{sr}<n\left(\gamma_{rd_{1}}\right)\right)\right]\}, \end{array}$$

where $l_{1}$, $m(\gamma_{sd_{1}})$, *n*, and $\gamma^{*}$ are as defined in Equation (38). Here, we restate them for the coherence of the discussion:

(A18) $$\begin{array}{cc} l_{1} & =\frac{2^{\tau_{1}}-1}{\alpha }, \end{array}$$

(A19) $$\begin{array}{cc} m(\gamma_{sd_{1}}) & =\frac{2^{\tau_{1}}-1}{\alpha }-\gamma_{sd_{1}}, \end{array}$$

(A20) $$\begin{array}{cc} n(\gamma ) & =\frac{\left(\gamma +1\right)m\left(\gamma_{sd_{1}}\right)}{\gamma -m(\gamma_{sd_{1}})},\gamma =\gamma_{sr},\gamma_{rd_{1}}, \end{array}$$

(A21) $$\begin{array}{cc} \gamma^{*} & =m\left(\gamma_{sd_{1}}\right)+\sqrt{{\left[m\left(\gamma_{sd_{1}}\right)\right]}^{2}+m\left(\gamma_{sd_{1}}\right).} \end{array}$$

In addition, $O_{2,d_{1}}$ can be expressed in the same form as in Equation (A17), with the difference that

(A22) $$\begin{array}{cc} l_{1} & =\frac{2^{\tau_{2}}-1}{1-\alpha 2^{\tau_{2}}}, \end{array}$$

(A23) $$\begin{array}{cc} m(\gamma_{sd_{1}}) & =\frac{2^{\tau_{2}}-1-\frac{\bar{\alpha }\gamma_{sd_{1}}}{1+\alpha \gamma_{sd_{1}}}}{\bar{\alpha }-\alpha \left(2^{\tau_{2}}-1-\frac{\bar{\alpha }\gamma_{sd_{1}}}{1+\alpha \gamma_{sd_{1}}}\right)}.\;\; \end{array}$$

For $O_{2,d_{1}}$ to be a subevent of $O_{1|2,d_{1}}$, an obvious condition that needs to be satisfied is that

$$\frac{2^{\tau_{2}}-1}{1-\alpha 2^{\tau_{2}}}\leq \frac{2^{\tau_{1}}-1}{\alpha }.$$

With some algebraic manipulations, it can be proved that the above condition is true only when $\alpha \leq \alpha^{max}$ under the assumption that $0<\alpha <\frac{1}{2^{\tau_{2}}}$. To proceed, we assumed that $\alpha \leq \alpha^{max}$. An interesting observation is that both n(γ) and $\gamma^{*}$ are monotonously increasing function of $m(\gamma_{sd_{1}})$.

Define

(A24) $$y\left(\gamma_{sd_{1}}\right)=\frac{2^{\tau_{1}}-1}{\alpha }-\gamma_{sd_{1}}-\frac{2^{\tau_{2}}-1-\frac{\bar{\alpha }\gamma_{sd_{1}}}{1+\alpha \gamma_{sd_{1}}}}{\bar{\alpha }-\alpha \left(2^{\tau_{2}}-1-\frac{\bar{\alpha }\gamma_{sd_{1}}}{1+\alpha \gamma_{sd_{1}}}\right)}.$$

It is intuitive to see that $O_{2,d_{1}}$ will be a subevent of $O_{1|2,d_{1}}$ if $y(\gamma_{sd_{1}})\geq 0$ is true for all $\gamma_{sd_{1}}<l_{1}$. In order to find the minimal $y(\gamma_{sd_{1}})$, expression Equation (A24) is differentiated with respect to $\gamma_{sd_{1}}$. The $\gamma_{sd_{1}}$ value that minimizes $y(\gamma_{sd_{1}})$ is

$$\gamma_{sd_{1}}^{*}=\frac{2^{\tau_{2}}-1}{\bar{\alpha }+1-\alpha 2^{\tau_{2}}}.$$

Obviously, $\gamma_{sd_{1}}^{*}<l_{1}$. Correspondingly,

$$y\left(\gamma_{sd_{1}}^{*}\right)=\frac{\left(2^{\tau_{1}}+1\right)\left[2-\alpha \left(2^{\tau_{2}}+1\right)\right]}{\alpha (\bar{\alpha }+1-\alpha 2^{\tau_{2}})}.$$

It can be validated by some manipulations that $y(\gamma_{sd_{1}}^{*})$ is nonnegative under the assumption that $\alpha \leq \alpha^{max}$.

In conclusion, with the AF broadcast transmission protocol, message $x_{2}$ is degraded to $x_{1}$ (from the decoding sense) if and only if the condition $\alpha \leq \alpha^{max}$ is satisfied.

## Appendix D. Outage Event of Conventional AF Relaying

Here, we characterize the outage event of a conventional AF protocol in a more intuitive format, such that the analytic outage expression can be easily written. We use $N_{d_{1}}$ as an example and the case of $N_{d_{2}}$ can be dealt with similarly. As in [7], the outage event of $N_{d_{1}}$ is given as

(A25) $$\gamma_{sd_{1}}+\frac{\gamma_{sr}\gamma_{rd_{1}}}{\gamma_{sr}+\gamma_{rd_{1}}+1}<2^{\tau_{1}}-1.$$

We define

(A26) $$t=m\left(\gamma_{sd_{1}}\right)=2^{\tau_{1}}-1-\gamma_{sd_{1}},$$

$$\gamma^{*}\left(\gamma_{sd_{1}}\right)=m\left(\gamma_{sd_{1}}\right)+\sqrt{{m(\gamma_{sd_{1}})}^{2}+m\left(\gamma_{sd_{1}}\right)}.$$

Then, we consider the event defined in Equation (A25), which can be equivalently expressed as

(A27) $$\left[\gamma_{sd_{1}}<2^{\tau_{1}}-1\right]⋂\left[\frac{\gamma_{sr}\gamma_{rd_{1}}}{\gamma_{sr}+\gamma_{rd_{1}}+1}<2^{\tau_{1}}-1-\gamma_{sd_{1}}\right].$$

The inequality

(A28) $$\frac{\gamma_{sr}\gamma_{rd_{1}}}{\gamma_{sr}+\gamma_{rd_{1}}+1}<t,$$

∀t>0, has the following two equivalent expressions:

(A29) $$\begin{array}{cc} \gamma_{rd_{1}}\left(\gamma_{sr}-t\right) & <(\gamma_{sr}+1)t, \end{array}$$

(A30) $$\begin{array}{cc} \gamma_{sr}\left(\gamma_{rd_{1}}-t\right) & <(\gamma_{rd_{1}}+1)t. \end{array}$$

It is obvious from Equations (A29) and (A30) that the inequality in Equation (A28) occurs when either of $\gamma_{sr}$ and $\gamma_{rd_{1}}$ is smaller than *t*, then we have $A_{1}$ in Equation (A31) (provided that $2^{\tau_{1}}-1-\gamma_{sd_{1}}>0$):

(A31) $$A_{1}\equiv \left[\gamma_{sr}\leq m\left(\gamma_{sd_{1}}\right)\right],⋃\left[\gamma_{rd_{1}}\leq m\left(\gamma_{sd_{1}}\right)\right],\;\;\;$$

Furthermore, we consider the case $\gamma_{sr}>t$ and $\gamma_{rd_{1}}>t$. In this case, the inequalities in Equations (A29) and (A30) convert to the following two events

(A32) $$\begin{array}{c} \left[\gamma_{sr}>t⋂\gamma_{sr}<\gamma_{rd_{1}}<\frac{(\gamma_{sr}+1)t}{\gamma_{sr}-t}\right]⋃\left[t<\gamma_{rd_{1}}<\min\left(\gamma_{sr},\frac{(\gamma_{sr}+1)t}{\gamma_{sr}-t}\right)\right] \end{array}$$

(A33) $$\begin{array}{c} \left[\gamma_{rd_{1}}>t⋂\gamma_{rd_{1}}<\gamma_{sr}<\frac{(\gamma_{rd_{1}}+1)t}{\gamma_{rd_{1}}-t}\right]⋃\left[t<\gamma_{sr}<\min\left(\gamma_{rd_{1}},\frac{(\gamma_{rd_{1}}+1)t}{\gamma_{rd_{1}}-t}\right)\right] \end{array}$$

respectively, the *intersection* of which forms the event defined by Equation (A28), which can be further expressed as an union of two disjoint events based on the relationship between $\gamma_{sr}$ and $\gamma_{rd_{1}}$ as is shown in Equations (A32) and (A33). First, consider if $\gamma_{sr}<\gamma_{rd_{1}}$, the intersection of Equations (A32) and (A33) results in the following event

(A34) $$\begin{array}{c} \left[\gamma_{sr}<\gamma_{rd_{1}}<\frac{(\gamma_{sr}+1)t}{\gamma_{sr}-t}\right]⋂\left[t<\gamma_{sr}<\min\left(\gamma_{rd_{1}},\frac{(\gamma_{rd_{1}}+1)t}{\gamma_{rd_{1}}-t}\right)\right]. \end{array}$$

Since $\gamma_{sr}<\gamma_{rd_{1}}$, it can be simply verified that $\frac{(\gamma_{sr}+1)t}{\gamma_{sr}-t}<\frac{(\gamma_{rd_{1}}+1)t}{\gamma_{rd_{1}}-t}$, ∀t>0 through the general discussion on monotonic functions. Then, we know that $\gamma_{rd_{1}}$ is smaller than $\frac{(\gamma_{rd_{1}}+1)t}{\gamma_{rd_{1}}-t}$. Thus, we obtain the valid range of $\gamma_{sr}$ as $t<\gamma_{sr}<\frac{(\gamma_{sr}+1)t}{\gamma_{sr}-t}$. With the inequality $\gamma_{sr}<\frac{(\gamma_{sr}+1)t}{\gamma_{sr}-t}$ and the fact that $\gamma_{sr}\geq 0$, we have $\gamma_{sr}<t+\sqrt{t^{2}+t}$. From all above discussions, Equation (A34) converts to

(A35) $$\begin{array}{c} \left[t<\gamma_{sr}<t+\sqrt{t^{2}+t}\right]⋂\left[\gamma_{sr}<\gamma_{rd_{1}}<\frac{(\gamma_{sr}+1)t}{\gamma_{sr}-t}\right]. \end{array}$$

By using $t=2^{\tau_{1}}-1-\gamma_{sd_{1}}$, Equation (A36) is obtained:

(A36) $$\begin{array}{cc} A_{2}\equiv & \left[m\left(\gamma_{sd_{1}}\right)<\gamma_{sr}<\gamma^{*}\left(\gamma_{sd_{1}}\right)\right]\\ & \;⋂\left[\gamma_{sr}<\gamma_{rd_{1}}<\frac{\left(\gamma_{sr}+1\right)m\left(\gamma_{sd_{1}}\right)}{\gamma_{sr}-m\left(\gamma_{sd_{1}}\right)}\right]. \end{array}$$

A similar discussion can be conducted for the case of $\gamma_{sr}\geq \gamma_{rd_{1}}$ and $A_{3}$ in Equation (A37) can be obtained. Details on this are omitted here for the sake of brevity:

(A37) $$\begin{array}{cc} A_{3}\equiv & \left[m\left(\gamma_{sd_{1}}\right)<\gamma_{rd_{1}}<\gamma^{*}\left(\gamma_{sd_{1}}\right)\right]\\ & \;⋂\left[\gamma_{rd_{1}}<\gamma_{sr}<\frac{\left(\gamma_{rd_{1}}+1\right)m\left(\gamma_{sd_{1}}\right)}{\gamma_{rd_{1}}-m\left(\gamma_{sd_{1}}\right)}\right]. \end{array}$$

In conclusion, the outage event of $Nd_{1}$ can be expressed as

(A38) $$\left[\gamma_{sd_{1}}<2^{\tau_{1}}-1\right]⋂\left[\underset{i=1,2,3}{⋃}A_{i}\right],$$

with $A_{1}$,$A_{2}$,$A_{3}$ are defined in Equations (A31), (A36) and (A37), respectively.

Thus, the outage probabilities of conventional DF relaying with orthogonal multiplexing at $N_{d_{1}}$ can be calculated as Equations (49)–(52).

## References

[1] Han G., Liu L., Zhang W., Zhang W. (2018). A hierarchical jammed-area mapping service for ubiquitous communication in smart communities. IEEE Commun. Mag..

[2] Han G., Yang X., Liu L., Zhang W. (2017). A joint energy replenishing and data collection algorithm in wireless rechargeable sensor networks. IEEE Internet Thing J..

[3] Han G., Yang X., Liu L., Guizani M., Zhang W. (2017). A disaster management-oriented path planning for mobile anchor-based localization in wireless sensor networks. IEEE Trans. Emerg. Top. Comput..

[4] Wang X., Yang T., Wortmann M., Shi P., Hattermann F., Lobanova A., Aich V. (2017). Analysis of multi-dimensional hydrological alterations under climate change for four major river basins in different climate zones. Clim. Chang..

[5] Yang T., Cui T., Xu C.Y. (2017). Development of a new IHA method for impact assessment of climate change on flow regime. Glob. Planet. Chang..

[6] Sendonaris A., Erkip E., Aazhang B. (2003). User cooperation diversity—Part I: System description. IEEE Trans. Commun..

[7] Laneman J.N., Tse D.N.C., Wornell G.W. (2004). Cooperative diversity in wireless networks: Efficient protocols and outage behavior. IEEE Trans. Inf. Theory.

[8] Khormuji M.N., Larsson E.G. (2009). Cooperative transmission based on decode-and-forward relaying with partial repetition coding. IEEE Trans. Wirel. Commun..

[9] Liang Y., Kramer G. (2007). Rate regions for relay broadcast channels. IEEE Trans. Inf. Theory.

[10] Bross S.I. (2009). On the discrete memoryless partially cooperative relay broadcast channel and the broadcast channel with cooperating decoders. IEEE Trans. Inf. Theory.

[11] Behboodi A., Piantanida P. (2013). Cooperative strategies for simultaneous and broadcast relay channels. IEEE Trans. Inf. Theory.

[12] Ding Z., Leung K.K., Goeckel D.L., Towsley D. (2010). Cooperative transmission protocols for wireless broadcast channels. IEEE Trans. Wirel. Commun..

[13] Lo E.S., Letaief K.B. (2011). Design and outage performance analysis of relay assisted two way wireless communications. IEEE Trans. Commun..

[14] Maham B., Hjrungnes A., Narasimhan R. (2011). Energy-efficient space-time coded cooperation in outage-restriced multihop wireless networks. IEEE Trans. Commun..

[15] Tse D., Viswanath P. (2005). Fundamentals of Wireless Communication.

[16] Goparaju A.K. (2005). Superposition Coding Based Co-Operative Diversity Schemes. Master’s Thesis.

[17] Men J.J., Ge J.H., Zhang C.S. (2017). Performance analysis of nonorthogonal multiple access for relaying networks over Nakagami-m fading channels. IEEE Trans. Veh. Technol..

[18] Shi S.L., Yang L.X., Zhu H. (2016). Outage balancing in downlink nonorthogonal multiple access with statistical channel state information. IEEE Trans. Wirel. Commun..

[19] Shi S.L., Yang L.X., Zhu H. (2015). Pairwise transmission using superposition coding for relay-assisted downlink communications. IEEE Trans. Wirel. Commun..

[20] Lv L., Chen J., Ni Q., Ding Z. (2017). Design of cooperative non-orthogonal multicast cognitive multiple access for 5G systems: User scheduling and performance analysis. IEEE Trans. Commun..

[21] Zhong C.J., Zhang Z.Y. (2016). Non-orthogonal multiple access with cooperative full-duplex relaying. IEEE Commun. Lett..

[22] Kim J.B., Lee I.H. (2015). Non-orthogonal multiple access in coordinated direct and relay transmission. IEEE Commun. Lett..

[23] Steiner A., Shamai S. (2006). Single-user broadcasting protocols over a two-hop relay fading channel. IEEE Trans. Inf. Theory.

[24] Zhang Y., Ma Y., Tafazolli R. (2010). Power allocation for bidirectional af relaying over rayleigh fading channels. IEEE Commun. Lett..

[25] Louie R.H.Y., Li Y., Vucetic B. (2010). Practical physical layer network coding for two-way relay channels: Performance analysis and comparison. IEEE Trans. Wirel. Commun..

